# Impairment of *PARK14*-dependent Ca^2+^ signalling is a novel determinant of Parkinson's disease

**DOI:** 10.1038/ncomms10332

**Published:** 2016-01-12

**Authors:** Qingde Zhou, Allen Yen, Grzegorz Rymarczyk, Hirohide Asai, Chelsea Trengrove, Nadine Aziz, Michael T. Kirber, Gustavo Mostoslavsky, Tsuneya Ikezu, Benjamin Wolozin, Victoria M. Bolotina

**Affiliations:** 1 Department of Medicine, Boston University School of Medicine, Boston, Massachusetts 02118, USA; 2 Department of Pharmacology and Experimental Therapeutics, Boston University School of Medicine, Boston, Massachusetts 02118, USA; 3 Center for Regenerative Medicine, Boston University School of Medicine, Boston, Massachusetts 02118, USA

## Abstract

The etiology of idiopathic Parkinson's disease (idPD) remains enigmatic despite recent successes in identification of genes (*PARKs*) that underlie familial PD. To find new keys to this incurable neurodegenerative disorder we focused on the poorly understood *PARK14* disease locus (*Pla2g6* gene) and the store-operated Ca^2+^ signalling pathway. Analysis of the cells from idPD patients reveals a significant deficiency in store-operated PLA2g6-dependent Ca^2+^ signalling, which we can mimic in a novel B6.Cg-*Pla2g6*^*ΔEx2-VB*^ (PLA2g6 ex2^KO^) mouse model. Here we demonstrate that genetic or molecular impairment of PLA2g6-dependent Ca^2+^ signalling is a trigger for autophagic dysfunction, progressive loss of dopaminergic (DA) neurons in substantia nigra pars compacta and age-dependent L-DOPA-sensitive motor dysfunction. Discovery of this previously unknown sequence of pathological events, its association with idPD and our ability to mimic this pathology in a novel genetic mouse model opens new opportunities for finding a cure for this devastating neurodegenerative disease.

## Abstract (web summary)

PLA2g6 regulates store-operated Ca^2+^ entry and is linked to Parkinson's disease. Here, Zhou *et al* find faulty PLA2g6-dependent Ca^2+^ signaling in idiopathic PD patients, and show that its impairment triggers autophagic dysfunction and loss of dopaminergic neurons in a new PLA2g6 ex2KO mouse model.

## Graphical abstract

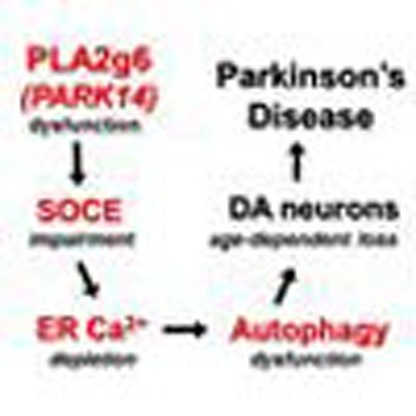

Genetic mutations in specific genes (*PARKs*) are thought to be the primary risk factors for familial Parkinson's disease (fPD), which comprises about 10% of all Parkinson's disease (PD) cases. Several *PARKs* have been linked to autophagic dysfunction, mitochondrial dysfunction, α-synuclein aggregation and other cellular defects, which are now viewed as hallmarks of human PD^1^,^2^,^3^,^4^. However, the vast majority of PD cases are idiopathic, occurring on ageing with no distinct triggers or clear underlying mechanisms. The aetiology of idiopathic PD (idPD) remains obscure, suggesting that not all pathological mechanisms underlying this disease may have been accounted for.

To find new triggers and signalling pathways that can lead to age-dependent idPD, we focused on the store-operated Ca^2+^ signalling and the poorly understood disease locus *PARK14*, which was identified as the *Pla2g6* gene^5^ encoding Ca^2+^-independent phospholipase A2 group 6 (PLA2g6 or iPLA_2_β). Distinct mutations in *Pla2g6* gene (*PARK14*) were found to be associated with familial Parkinsonism^6^,^7^,^8^,^9^,^10^,^11^, although the underlying mechanism and the role of PLA2g6 in idPD remain unclear. The PLA2g6 is a multifaceted enzyme that is best known for its catalytic function, which was linked to phospholipid remodelling in cells (for review see ref. 12). The loss of the catalytic activity of PLA2g6 was associated with infantile neuroaxonal dystrophy (INAD), and results in early death in humans and in mouse models^13^,^14^,^15^,^16^,^17^,^18^,^19^. In contrast to INAD mutations, PD-associated mutations in PLA2g6 were reported not to affect its catalytic activity^20^. The question remains open as to which cellular function of PLA2g6 could be involved in human PD, and may be responsible for a PD-like phenotype that would appear later with ageing.

Earlier, we discovered^21^,^22^,^23^,^24^,^25^,^26^ and others confirmed^27^,^28^,^29^,^30^,^31^ that PLA2g6 plays an important role in the activation of endogenous store-operated Ca^2+^ entry (SOCE). Notably, besides Orai1 (store-operated plasma membrane Ca^2+^ channel) and STIM1 (Ca^2+^ sensor in endoplasmic reticulum (ER)), PLA2g6 was identified in an RNAi screen as one of the essential components of endogenous SOCE (supplementary material in ref. 32). It is well established that SOCE is activated on depletion of ER Ca^2+^ stores (for review see refs 33, 34, 35), and is crucial for their timely refilling in a wide variety of cell types. However, the role of store-operated Ca^2+^ signalling in dopaminergic (DA) neurons and PD remains largely unknown.

This study was designed to explore the origins of human age-dependent PD from the new perspective of* PARK14* and the store-operated Ca^2+^ signalling. We tested a novel idea that impairment of the store-dependent Ca^2+^ signalling function of *PARK14* could be a previously unknown determinant of idPD and *PARK14* form of fPD. Using a wide array of genetic, molecular, cellular, imaging, behaviour and other approaches for *in vivo* and *in vitro* studies in human cells and new mouse model, we discovered a major source for cellular vulnerability that can be associated with premature death of DA neurons and age-dependent PD.

## Results

### SOCE and PLA2g6 are impaired in idPD and fPD^R747W^ patients

One of the requirements for Ca^2+^ signalling studies is the live-cell preparation. Because live DA neurons cannot be extracted from the midbrain of PD patients, human primary skin fibroblasts (hPSFs) provide an alternative cellular model representing PD and control patients, which proved to be instrumental for the studies of human neurodegeneration. To determine if idPD may be associated with changes in store-operated Ca^2+^ signalling, primary skin fibroblasts from control donors and idPD patients were obtained from the NINDS Cell Line Repository (http://ccr.coriell.org/ninds; Supplementary Table 1), and used in our studies. The idPD group was represented by aged (62±5 years old) male Caucasian idPD patients with no documented mutations in the PARK genes and no family history of PD. The group of neurological control donors was represented by aged (68±8 years old) Caucasian males with no symptoms or family history of PD.

**Figure 1:**
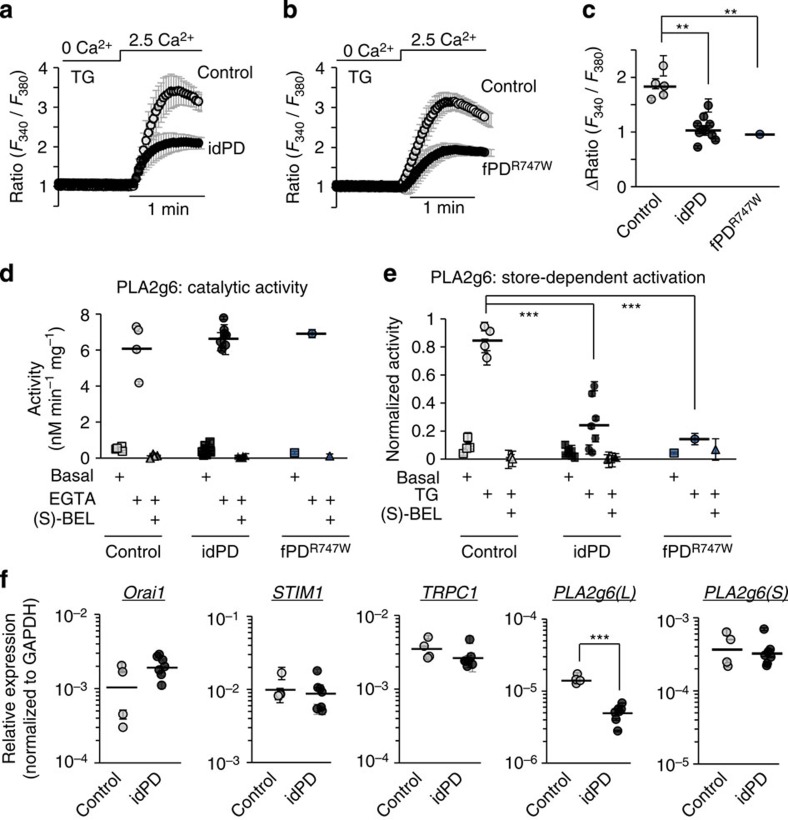
Deficiency in PARK14 and store-operated Ca^2+^ signalling in human primary skin fibroblasts from patients with idPD and with familial PLA2g6^R747W^ mutation (fPD^R747W^). (**a**,**b**) Representative traces show SOCE on Ca^2+^ addition in TG-pretreated (5 μM for 20 min) fibroblasts from control, idPD patients (in **a**) and fPD^R747W^ patient (in **b**): each trace show Ca^2+^ influx (average Ratio (*F*_340_/*F*_380_)±s.d.) in a group of 10–20 individual cells measured simultaneously; full traces are shown in Supplementary Fig. 1. (**c**) Comparative analysis of SOCE in fibroblasts from control idPD and fPD^R747W^ patients. Data for each patient show the average±s.e. from at least three independent experiments, with up to 120 cells analysed for each patient (see Supplementary Fig. 1 for details). (**d**) Catalytic PLA2g6 activity in homogenates of fibroblasts from control, idPD and fPD^R747W^ patients: summary results show average activity (±s.e.) from three repetitions under basal conditions, after activation in the presence of 10 mM EGTA and after inhibition with 25 μM of (S)-BEL (see Supplementary Fig. 2a for details). (**e**) Activation of PLA2g6 by TG-induced depletion of Ca^2+^ stores in intact fibroblasts from control, idPD and fPD^R747W^ patients: summary results show average activity (±s.e.) from three repetitions under basal conditions, after activation by TG (5 μM for 10 min) and after inhibition with (S)-BEL (see Supplementary Fig. 2b for details). (**f**) Relative expression of *Orai1*, *STIM1*, *TRPC1*, *PLA2g6(L)* and *PLA2g6(S)* in fibroblasts from control and idPD patients: summary results of quantitative reverse transcription–PCR analysis normalized to *GAPDH* for each sample, with group averages shown by the horizontal line (see Supplementary Fig. 3 for details). **P<0.01, ***P<0.001.

Analysis of live primary skin fibroblasts (P3–5) from idPD and control donors revealed a significant deficit in endogenous SOCE in the cells from all idPD patients tested (Fig. 1a,c and Supplementary Fig. 1): on average, there was more than 40% reduction in thapsigargin (TG)-induced Ca^2+^ influx in idPD patients compared with the control group. SOCE in both groups was highly sensitive to diethylstilbestrol (inhibitor of PLA2g6-dependent Orai1-mediated SOCE^36^,^37^): 10 μM of diethylstilbestrol produced 80±1% and 82±4% inhibition of TG-induced Ca^2+^ influx in control and idPD groups, respectively.

Importantly, we found that the cells from a patient with fPD associated with R747W mutation in *PARK14*/PLA2g6 (fPD^R747W^) have a similar deficit in SOCE (Fig. 1b,c and Supplementary Fig. 1).

To understand which PLA2g6 function may be associated with human PD, catalytic activity and store-dependent activation of PLA2g6 was analysed and compared in the cells from control, idPD and fPD^R747W^ patients. We found that activation of PLA2g6 by TG-induced Ca^2+^ store depletion^26^ seen in control donors is significantly impaired in the cells from idPD and fPD^R747W^ patients, while the catalytic activity of PLA2g6 is the same (Fig. 1d,e and Supplementary Fig. 2a,b). The specificity of PLA2g6 activity in both cases was confirmed by its inhibition with S-BEL, a chiral-specific suicidal substrate that discriminates PLA2g6 from all other phospholipases^38^.

Notably, the analysis of the expression of several major components of SOCE revealed that SOCE deficiency in the cells from idPD patients was not associated with reduction in *Orai1*, *TRPC1*, *STIM1* or *STIM2* expression: Fig. 1f and Supplementary Figs 3 and 4 demonstrate no difference in mRNA levels for these genes in control and PD patients. In contrast, we found that the expression of *PLA2g6(L)* (a specific plasma membrane-associated splice variant of PLA2g6 (ref. 39)) is significantly reduced in idPD patients (Fig. 1f and Supplementary Fig. 3b), while expression of the *PLA2g6(S)* splice variant (which lacks exon 8b and encodes a cytosolic enzyme that was shown to be involved in lipid remodelling^12^) is the same, as in control donors.

Thus, primary skin fibroblasts from idPD and fPD^R747W^ patients share a striking deficiency in store-dependent activation of PLA2g6, as well as significant impairment in endogenous SOCE.

### PLA2g6 ex2^KO^ mice mimic SOCE deficiency in idPD patients

To determine if and how the defects in the store-dependent activation of PLA2g6 and impaired SOCE could be translated into the age-dependent PD, we sought a mouse model with targeted impairment of these specific cellular functions. Currently existing transgenic PLA2g6 mouse models^13^,^14^,^15^,^16^,^17^,^18^ appear to be unsuitable for PD studies, as they have impaired catalytic activity of PLA2g6, which we found to be unaffected in idPD patients. Moreover, PD pathology develops later in life, while the loss of catalytic activity of PLA2g6 is associated with INAD and early death in mice and humans.

**Figure 2:**
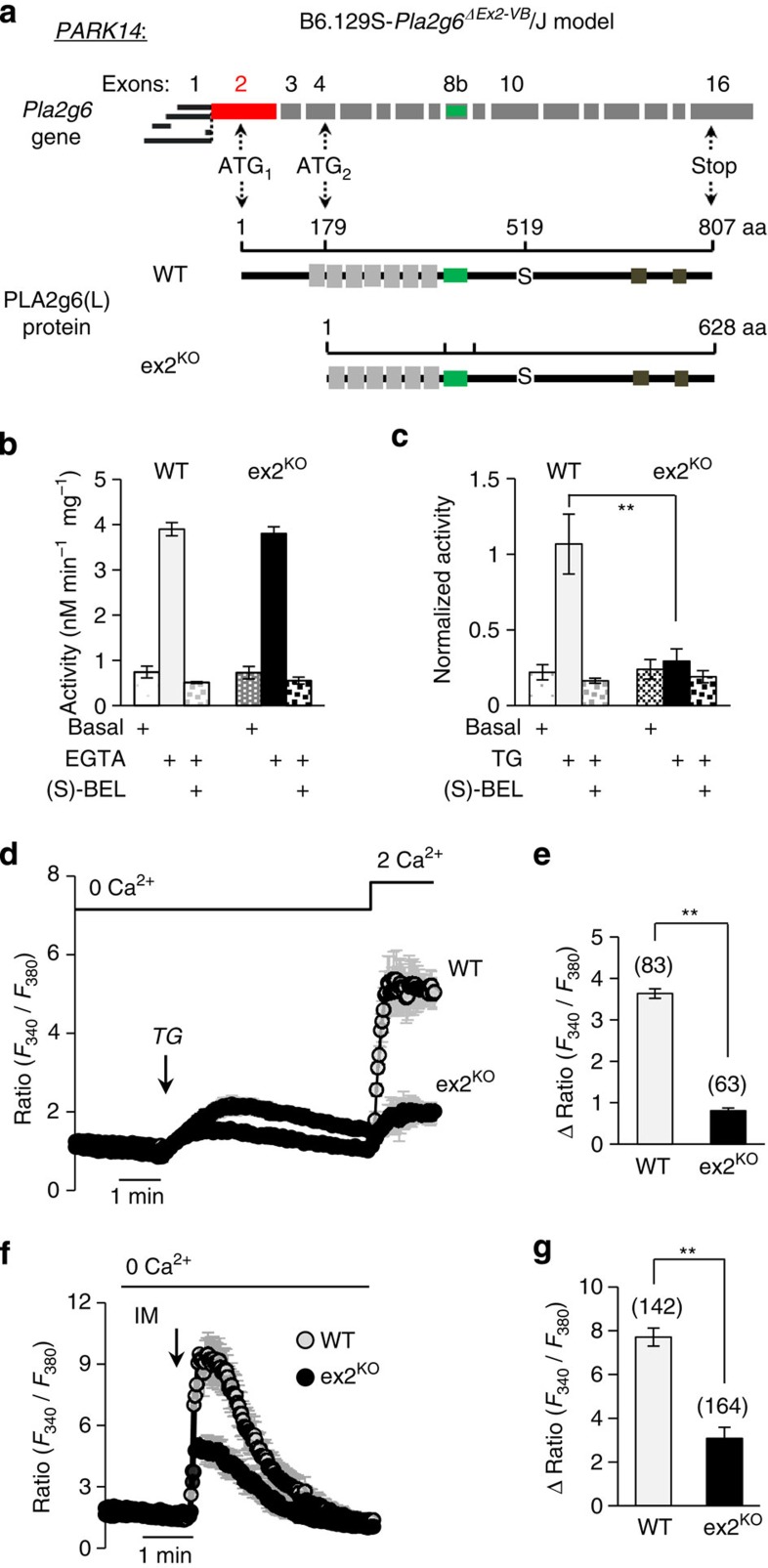
New *PARK14* (PLA2g6) ex2^KO^ mouse model mimics idPD-associated deficiency in store-operated PLA2g6-dependent Ca^2+^ signalling. (**a**) Schematic illustration of *Pla2g6* (*PARK14*) gene with exons and ATG sites; below, corresponding full-length (L) variant of WT PLA2g6 protein and N-terminal truncated protein in ex2^KO^ mouse in which exon 2 (in red) was genetically deleted (Supplementary Figs 5–9). (**b**) Catalytic activity of PLA2g6 in homogenates of MEFs from WT and ex2^KO^ mice: summary results show average activity (±s.e.) from three repetitions under basal conditions, after activation in the presence of 10 mM EGTA and after inhibition with 25 μM (S)-BEL. (**c**) Activation of PLA2g6 by TG-induced depletion of Ca^2+^ stores in intact MEFs from WT and ex2^KO^ mice: summary results show average activity (±s.e. from three repetitions) under basal conditions, after activation by TG (5 μM for 10 min) and after inhibition with (S)-BEL. (**d**) Impairment of TG-induced SOCE in the ex2^KO^ MEFs: representative traces show Ca^2+^ response to TG (5 μM) application in the absence of extracellular Ca^2+^, followed by SOCE on Ca^2+^ addition in WT and ex2^KO^ cells. Each trace shows Ca^2+^ responses (average±s.d.) in a group of 10–20 individual cells measured simultaneously. (**e**) Summary data show the differences in the peak SOCE in the WT and ex2^KO^ cells: average±s.e. from 3–6 independent experiments per each condition. (**f**) Representative traces (average±s.d.) show ionomycin (IM, 1 μM)-induced Ca^2+^ release from intracellular stores (in the presence of extracellular EGTA) in WT and ex2^KO^ MEFs. (**g**) Summary data from experiments like in **f** show peak Ca^2+^ release (average±s.e.) from 3–6 independent experiments. The numbers of cells summarized for each condition is specified above the bars; **P<0.01.

To create a new model suitable for PD studies, we targeted PLA2g6 in a different manner. We hypothesized that genetic deletion of the N terminus of PLA2g6 (Fig. 2a) should not affect its catalytic activity (encoded by S^519^-containing catalytic domain in the C terminus), but may impair PLA2g6 activation by depleted stores, thus reproducing the specific deficiency that we found in idPD patients. To test this approach, a novel PLA2g6 ex2^KO^ mouse model was created (B6.Cg-*Pla2g6*^*ΔEx2-VB*^/J), in which exon 2 of *Pla2g6* gene was constitutively deleted (see Fig. 2a, Supplementary Figs 5–7 and Methods for details on generation and validation of this model). Deletion of the translation initiation ATG_1_ (coded by exon 2), did not affect expression of (L) and (S) splice variants of PLA2g6 (Supplementary Fig. 8), and did not lead to the loss of PLA2g6 protein: the presence of a cryptic ATG_2_ in exon 4 initiated translation and resulted in the expression of the truncated ex2^KO^ PLA2g6 protein that lacks the first 178 amino acids in the N terminus (Supplementary Fig. 9 and 10), while the rest of the molecule remained intact.

Analysis of the PLA2g6 activity in mouse embryonic fibroblasts (MEFs) from wild-type (WT) and ex2^KO^ animals revealed that genetic truncation of the N terminus did not affect its catalytic activity (Fig. 2b), but resulted in the loss of PLA2g6 activation by TG-induced depletion of the stores (Fig. 2c). Thus, similar to the cells from idPD and fPD^R747W^ patients, ex2^KO^ mice appear to have deficiency in the store-dependent activation of PLA2g6. It is important to emphasize that preserved catalytic activity of PLA2g6 clearly discriminates this new ex2^KO^ mouse model from other PLA2g6 models^13^,^14^,^15^,^16^,^17^,^18^, in which catalytic activity of this enzyme was genetically impaired.

To test if PLA2g6 ex2^KO^ mice could also mimic the defect of the store-operated Ca^2+^ signalling that we found in fibroblasts from idPD patients, endogenous SOCE was analysed and compared in MEFs from WT and ex2^KO^ animals. The results of these studies indicated that TG-induced SOCE is significantly impaired in MEFs from ex2^KO^ mice (Fig. 2d,e). Similarly, we found that TPEN (a low-affinity Ca^2+^ chelator that is known to mimic depletion of Ca^2+^ in ER stores^40^,^41^) activates PLA2g6-dependent (BEL-sensitive) SOCE in the cells from WT, but not from ex2^KO^ mice (Supplementary Fig. 11). Consistent with the physiological role of SOCE in refilling of ER Ca^2+^ stores, significant impairment of endogenous SOCE resulted in constitutive depletion of intracellular Ca^2+^ stores in ex2^KO^ cells, as indicated by more than 50% decrease in intracellular Ca^2+^ release caused by ionomycin (Fig. 2f,g). Passive Ca^2+^ release in response to TG application in the absence of extracellular Ca^2+^ (Fig. 2d) was also significantly decreased in ex2^KO^ cells (by 56±5%, *P*<0.01).

Importantly, primary skin fibroblasts from idPD patients also appear to have significant reduction in ionomycin-induced Ca^2+^ release: the ΔRatio (*F*_340_/*F*_380_) was 5.9±0.5 (*n*=7) in idPD and 8.5±0.9 (*n*=4) in control patients, respectively (*P*<0.05).

Therefore, PLA2g6 ex2^KO^ mice exhibit major functional deficiencies in store-operated PLA2g6-dependent Ca^2+^ signalling that mirrors the pathological cellular phenotype found in idPD and fPD^R747W^ patients (Fig. 1): MEFs from ex2^KO^ mice (i) retain normal catalytic activity of PLA2g6 (Fig. 2b), (ii) have a major defect in PLA2g6 activation by depleted stores (Fig. 2c), (iii) have a significant impairment of endogenous SOCE and (iv) show a significant depletion of intracellular Ca^2+^ stores (Fig. 2f,g). The PLA2g6 ex2^KO^ mouse model offers a unique tool to determine if and how such cellular deficiencies could lead to PD pathology.

### iPSC-derived DA neurons from PLA2g6 ex2^KO^ and WT mice

The role of SOCE in live DA neurons is obscure, so the next two questions were (i) whether SOCE is present in DA neurons and (ii) if DA neurons from PLA2g6 ex2^KO^ mice had similar defects in SOCE and ER Ca^2+^, as we found in fibroblasts. To assess the role of PLA2g6 and SOCE in live DA neurons, an induced pluripotent stem cell (iPSC) approach was used to create iPSC-derived A9 midbrain DA neurons^42^ from WT and PLA2g6 ex2^KO^ MEFs (see Methods and Supplementary Fig. 12).

**Figure 3:**
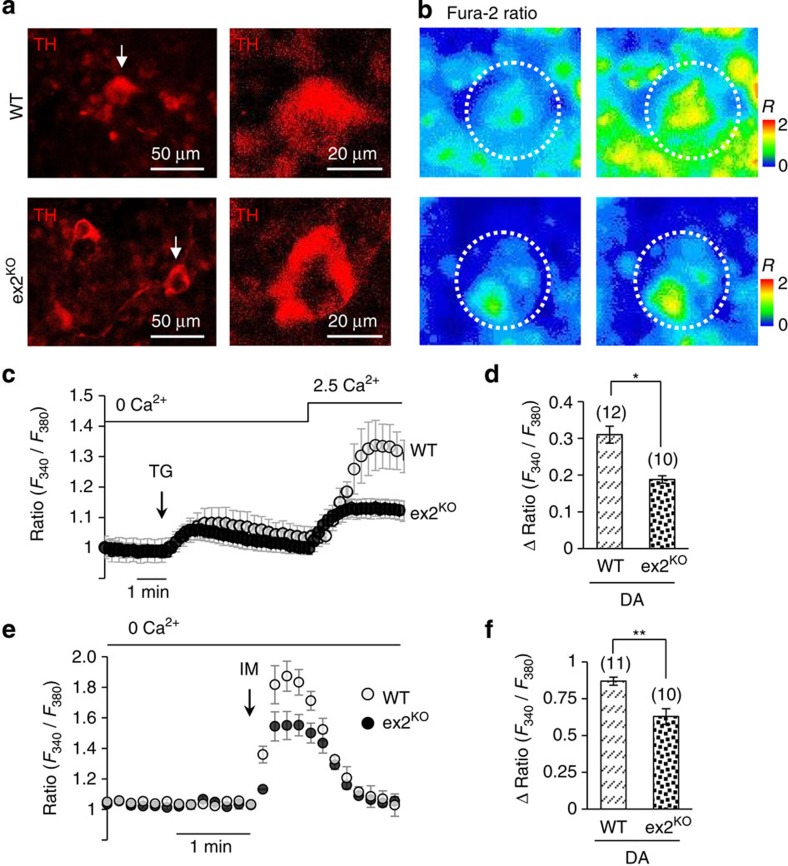
Store-operated Ca^2+^ signalling in iPSC-derived DA neurons from WT and ex2^KO^ mice. (**a**) Representative images of iPSC-derived A9 midbrain DA neurons positive for TH (TH+) from WT and PLA2g6 ex2^KO^ mice (see Supplementary Fig. 12 for more details); (**b**) images demonstrate Ca^2+^ changes due to SOCE in specific DA neurons outlined by dotted circles and shown by an arrow in **a**: images show Fura-2 Ratio (*F*_340_/*F*_380_) in individual TH+ neuron before (left) and after (right) Ca^2+^ addition to TG-pretreated cells, as shown in **c**. (**c**) TG-induced SOCE (average±s.d.) in individual iPSC-derived DA (TH+) neurons from WT and ex2^KO^ mice: traces show Ca^2+^ changes in response to TG (5 μM) application in the absence of extracellular Ca^2+^, followed by SOCE on Ca^2+^ addition. (**d**) Summary data comparing the peak SOCE (average±s.e.) in DA (TH+) neurons from WT and ex2^KO^ mice. (**e**) Ionomycin (IM; 100 nM)-induced Ca^2+^ release (average±s.d.) from intracellular stores in DA (TH+) neurons from WT and ex2^KO^ mice. (**f**) Summary data from experiments like in **f** show peak Ca^2+^ release (average±s.e.). The data represent the results from three independent experiments. The numbers of cells analysed for each condition is specified above the bars; *P<0.05, **P<0.01.

Live-cell Ca^2+^ imaging (Fig. 3a,b) revealed that iPSC-derived tyrosine hydroxylase-positive (TH+) DA neurons indeed have classical TG-induced Ca^2+^ responses (Fig. 3c), including passive release of Ca^2+^ from the stores and SOCE that could be detected on Ca^2+^ re-addition. These data present first evidence for SOCE in iPSC-derived DA neurons. Notably, the amplitude of SOCE in DA neurons appeared to be very small compared with SOCE in MEFs under the same experimental conditions: on Ca^2+^ addition to TG-treated cells ΔRatio was 0.31±0.18 in DA neurons (*n*=12) versus 2.39±0.16 (*n*=31) in MEFs. Similarly, ionomycin-induced Ca^2+^ release in DA neurons (Fig. 3e,f) also appeared to be significantly smaller than in MEFs: ΔRatio=0.87±0.03 (*n*=11) in DA neurons versus 2.66±0.21 (*n*=26) in MEFs. Small Ca^2+^ release was consistent with previously acknowledged low Ca^2+^ buffering capacity of intracellular Ca^2+^ stores in DA neurons of substantia nigra pars compacta (SNc)^43^.

Further, we found that SOCE and ionomycin-induced Ca^2+^ store release are significantly impaired in DA neurons from PLA2g6 ex2^KO^ mice (Fig. 3c–f), emulating the results obtained in MEF cells. Thus, impairment of PLA2g6 translates into significant loss of apparently limited store-operated Ca^2+^ signalling in DA neurons, which could make these cells particularly vulnerable to PLA2g6 and SOCE dysfunction.

### PLA2g6 ex2^KO^ mice develop age-dependent PD-like phenotype

**Figure 4:**
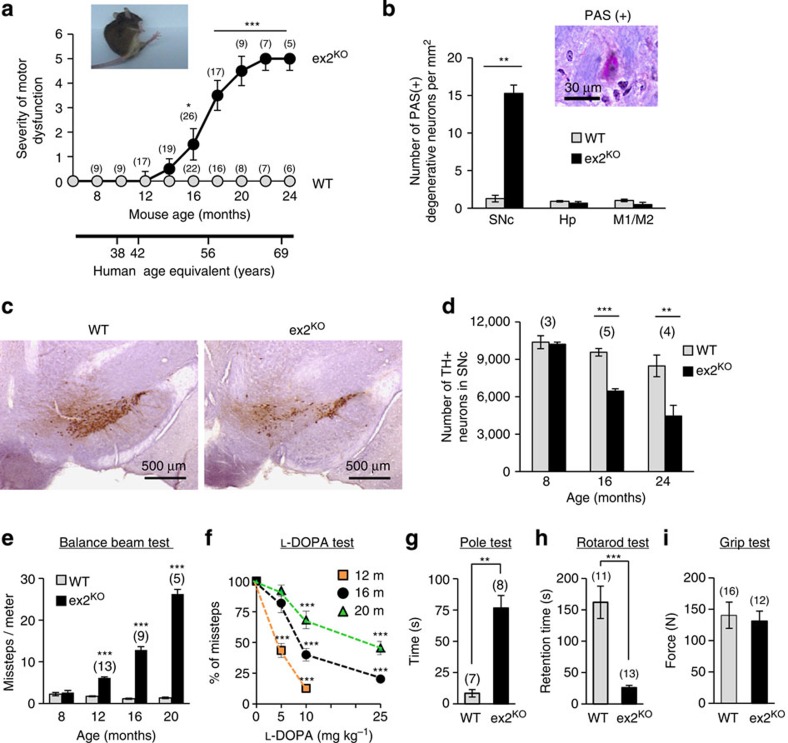
Progressive loss of DA neurons and age-dependent PD-like motor dysfunction in ex2^KO^ mice. (**a**) progressive motor dysfunction in ageing ex2^KO^, but not in WT mice (see Methods for details); human age equivalent is shown below; inset illustrates unstable gait of representative 18-month-old ex2^KO^ animal; numbers above each point represent the number of animals per each age group. (**b**) Analysis of PAS staining shows significant increase in the number of PAS(+) degenerative neurons in SNc, but not in hippocampus (Hp) or in M1/M2 motor cortex of the 16-month-old ex2^KO^ mice (average±s.e. from three pairs of age-matched animals). (**c**) Example of immunostaining of TH+ (brown) neurons in SNc in the brain from WT and ex2^KO^ littermates (16 months old); scale bar, 500 μm. (**d**) Summary data (average±s.e.) show progressive age-dependent reduction in the number of TH+ neurons in ex2^KO^ mice; the results of the blinded stereological analysis of SN area of the brain in the groups of age-matched WT and ex2^KO^ animals (for more details see Supplementary Fig. 13). (**e**–**i**) Behavioural studies of the age-matched groups of the WT and ex2^KO^ mice show: (**e**) progressive age-dependent increase in the number of missteps in the balance beam test; (**f**) age- and dose-dependent improvement of motor performance by L-DOPA in ex2^KO^ mice; relative change in the number of missteps in balance beam test made 1h after L-DOPA (5, 10 or 25 μM) administration versus control in the 12 (*n*=12)-, 16 (*n*=8)- and 20 (*n*=8)-month-old animals; (**g**) significant increase in time required for 16-month-old ex2^KO^ mice to reorient on the top of the pole; (**h**) significantly reduced rotarod performance of the 16- to 18-month-old ex2^KO^ mice; (**i**) no difference in the grip strength of the 16- to 18-month-old WT and ex2^KO^ mice. All data are mean±s.e.; numbers above each bar represent the number of animals tested per each group; **P<0.01, ***P<0.001.

Ageing is a primary risk factor for idPD^44^, and strikingly, all homozygous PLA2g6 ex2^KO^ mice (males and females) developed progressive age-dependent motor dysfunction (Fig. 4a) at an age range that aligns with that typical of idPD in humans. WT and heterozygous littermates remained normal throughout the same observation period, consistent with autosomal recessive inheritance of *PARK14* (PLA2g6)-associated fPD in humans. Analysis of the SNc area of the brain from aged animals revealed a significant increase in the number of degenerative periodic acid–Schiff (PAS)-positive neurons (Fig. 4b) in the ex2^KO^ animals, and Nissl staining suggested potential loss of DA neurons (Supplementary Fig. 13). The results of the blinded stereological analysis of SNc confirmed progressive age-dependent loss of TH+ DA neurons (Fig. 4c,d and Supplementary Figs 13 and 14): while the number of TH+ neurons was the same in 8-month-old ex2^KO^ and WT animals (consistent with no motor dysfunction in this preclinical stage), over 30% of DA neurons in SNc of ex2^KO^ mice was lost by 16 months (early clinical) and over 50% was lost by 24 months of age (late clinical stage). Notably, analysis of the hippocampus and M1/M2 areas of the temporal cortices revealed no signs of neurodegeneration in ex2^KO^ animals (Fig. 4b), demonstrating that motor dysfunction and DA neuronal loss in SNc was not the result of a widespread nonspecific neurodegeneration.

Progressive loss of DA neurons in SNc is known to be a major factor in motor dysfunction in human PD^45^. Similarly, ex2^KO^ animals developed a strong age-dependent PD-like motor dysfunction, which was validated using an array of the standard behavioural tests (Fig. 4e–i). The balance beam test (Fig. 4e) showed impairment of motor coordination and progressive age-dependent increase in the number of missteps made by ex2^KO^ mice, with no change in performance of ageing WT animals. Importantly, the DA nature of motor dysfunction was confirmed by the L-DOPA test (Fig. 4f): administration of L-DOPA markedly improved motor coordination of ex2^KO^ animals in age- and dose-dependent manner, which was similar to L-DOPA effects in humans^46^. Figure 4f shows that while the lowest dose of L-DOPA (5 mg per kg body weight) produced a marked improvement in the balance beam performance (60% reduction in the number of missteps) of the 12-month-old ex2^KO^ animals (early clinical stage), significantly higher doses were required to produce similar effects at more advanced clinical stages in 16- and 20-month-old animals. The pole (Fig. 4g) and rotarod tests (Fig. 4h) further confirmed significant PD-like motor dysfunction in ex2^KO^ animals. Grip test (Fig. 4i) showed no difference between WT and ex2^KO^ animals, thus demonstrating that motor dysfunction in ex2^KO^ animals is not caused by the loss of the strength in their limbs.

Thus, PLA2g6 ex2^KO^ mouse model presents progressive loss of DA neurons in SNc and age-dependent L-DOPA-sensitive PD-like motor dysfunction, which mimics idPD in ageing humans.

### Autophagic dysfunction in DA neurons of PLA2g6 ex2^KO^ mice

**Figure 5:**
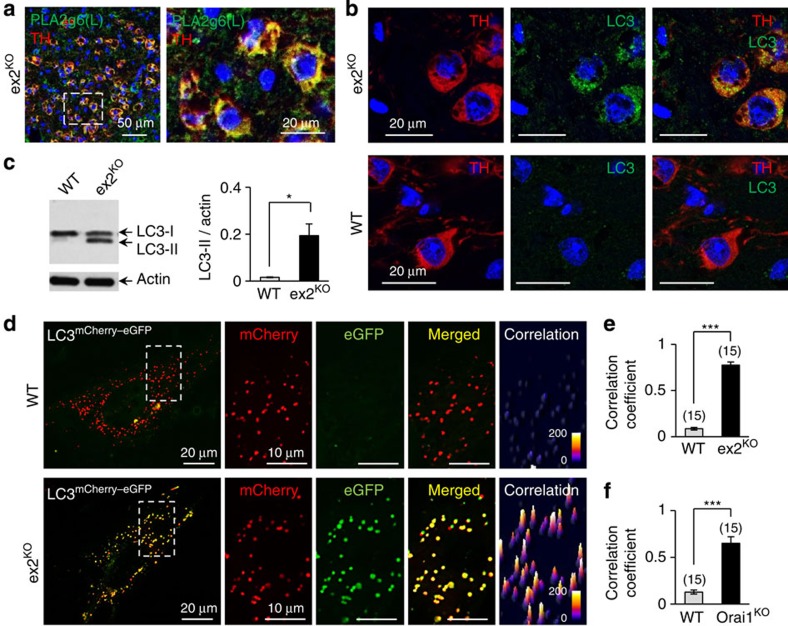
Autophagic dysfunction in ex2^KO^ mice. (**a**) Co-localization of TH (red) and PLA2g6(L) (green) in DA neurons in SNc of ex2^KO^ mice (see Supplementary Fig. 14 for details). Nuclei are stained with DAPI (blue). Image on the right shows magnified part of the image on the left (identified by dotted rectangle). (**b**) Representative images show LC3 aggregation in TH+ neurons of the ex2^KO^, but not WT brain: results of co-immunostaining for TH (red), LC3 (green) and DAPI (blue) in SNc area of the brain from 16-month-old ex2^KO^ and WT littermates (see Supplementary Fig. 15 for details). (**c**) Representative western blot (WB) and summary data (average±s.e.) show significant increase in LC3-II/actin ratio in tissue samples from the ex2^KO^ mice (*n*=3). Images have been cropped for presentation. Full-size images are shown in Supplementary Fig. 22. (**d**) Representative images of LC3^mCherry–eGFP^ (tandem mCherry (red)–eGFP (green)-tagged LC3) in live MEF cells from the WT and ex2^KO^ mice. Composite image of the whole cell is shown on the left, and magnified mCherry, eGFP and merged images on the right show the part of the cell identified by dotted rectangle. Far right image shows correlation map for red and green signals (see Supplementary Fig. 17 and Methods for details). (**e**) Summary data show increase in the correlation coefficient (in experiments like in **d**) in the cells from ex2^KO^ mice, compared with WT cells; bars show average (±s.e.) from three independent experiments. (**f**) Summary data like in **e** show significant increase in the correlation coefficient in the cells from Orai1^KO^ mice (for more details see Supplementary Fig. 18). *P<0.05, ***P<0.001.

The question arises, how does impairment of store-operated Ca^2+^ signalling lead to demise of DA neurons in SNc? More specifically, could disruption in store-operated Ca^2+^ signalling trigger some cellular pathology that is already established as a hallmark of human PD? Analysis of the SNc area of the brain in ex2^KO^ mice revealed that PLA2g6(L) protein is highly expressed in specific TH+ DA neurons (Fig. 5a and Supplementary Fig. 15), suggesting that DA neurons in SNc may have a particularly high demand for PLA2g6(L), and impairment of the PLA2g6-dependent SOCE function (Fig. 3) may be particularly stressful for these neurons. Closer analysis of TH+ neurons in SNc of PLA2g6 ex2^KO^ mice revealed that DA neurons experience significant autophagic dysfunction. Figure 5b and Supplementary Fig. 16 show that TH+ neurons in SNc in ex2^KO^ have significant accumulation of LC3 (microtubule-associated protein 1A/1B-light chain 3, established marker of autophagic flux), which is not found in WT animals. The increased autophagosome numbers in ex2^KO^ mice, which can result from impaired autophagic flux, was also manifested by a significant increase in the ratio of LC3-II/actin (Fig. 5c). Thus, *in vivo* DA neurons in the SNc of PLA2g6-deficient ex2^KO^ mice may experience marked autophagic dysfunction, which is one of the major hallmarks of human PD^47^,^48^,^49^,^50^,^51^.

### Causal link between PLA2g6, SOCE, ER Ca^2+^ and autophagy

To better understand a previously unknown association of PLA2g6-dependent Ca^2+^ signalling with autophagy, primary MEFs from WT and ex2^KO^ mice were used as a model for live-cell imaging and molecular rescue studies. Using a tandem tagged LC3^mCherry–eGFP^ as a marker of autophagic flow^52^ we confirmed significant autophagic dysfunction in the cells from ex2^KO^ animals. Image analysis of MEFs expressing LC3^mCherry–eGFP^ (Fig. 5d) revealed that in WT cells this marker successfully reaches lysosomes, where the eGFP (but not mCherry) signal is quenched by the high acidic environment, resulting in a loss of green fluorescent protein (GFP) fluorescence. In contrast, the enhanced GFP (eGFP) signal remained very prominent and spatially co-localized with mCherry in autophagosomes of ex2^KO^ cells. Correlation analysis of mCherry and eGFP fluorescence confirmed very significant differences between ex2^KO^ and WT cells: in ex2^KO^ cells, the fluorescent signals were strongly correlated (Fig. 5e) and the size of LC3-containing particles was larger (Supplementary Fig. 17) than in WT cells. Autophagic arrest in ex2^KO^ cells resembled the effects of prolonged TG treatment in WT MEFs (Supplementary Fig. 17), which was consistent with recent report^53^ of TG-induced autophagic arrest due to impairment of autophagosome fusion with lysosomes. There is also a possibility that fusion may occur, but lysosomal acidification may be defective.

Interestingly, a similar autophagic dysfunction was also produced by the targeted deletion of the Orai1 channel (Fig. 5f), which is a critical component of SOCE machinery located downstream from PLA2g6 (refs 25, 26). Supplementary Fig. 18 demonstrate that MEF cells from Orai1^KO^ mice^32^,^54^ have significant impairment of SOCE, depletion of ER Ca^2+^ stores and autophagic dysfunction, which closely mimic deficiencies in PLA2g6 ex2^KO^ cells. Thus, similar autophagic arrest can be produced by genetic deletion of Orai1, or impairing PLA2g6 activation, or inhibiting SERCA (Sarco/endoplasmic rReticulum Ca^2+^ ATPase)-dependent refilling of the stores with TG. Remarkably, while all these interventions cause depletion of ER Ca^2+^ stores, only Orai1^KO^ and ex2^KO^ inhibit SOCE, while TG activates it. Thus, depletion of Ca^2+^ stores (rather than simple loss of SOCE) seems to be a most likely trigger for autophagic dysfunction.

**Figure 6:**
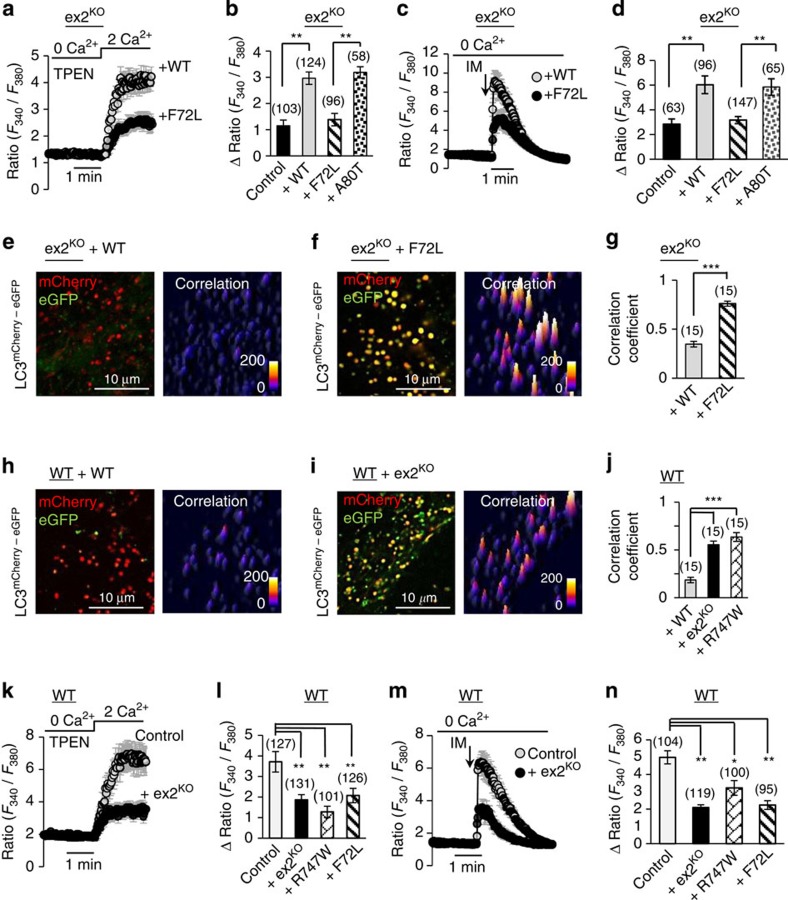
Causal relationship between PLA2g6-dependent Ca^2+^ signalling and autophagic dysfunction, and its relevance to human PD. (**a**–**g**) Rescue experiments in live ex2^KO^ MEF cells transfected with WT PLA2g6(L) or its PD-associated F72L mutant, or A80T mutant that does not have association with human PD. (**h**–**n**) Dominant-negative effects of PLA2g6(L) deficiency in live WT MEF cells transfected with empty vector (control) or PLA2g6(L) ex2^KO^ or one of two human PD-associated PLA2g6(L) mutants (F72L or R747W). (**a**,**b**,**k**,**l**), SOCE on Ca^2+^ addition to TPEN-pretreated cells (400 μM for 3 min); (**c**,**d**,**m**,**n**) ionomycin (IM, 1 μM)-induced intracellular Ca^2+^ store release; (**e**–**g**,**h**–**j**) autophagic flow visualized by LC3^mCherry–eGFP^ fluorescence (as in Fig. 4d–e) in live MEF cells. Representative Ca^2+^ traces show average±s.d. from 10–20 cells recorded simultaneously. All summary data (bars) show average±s.e. from 3–6 independent experiments per each condition; the number of cells for each condition is specified above the bars; *P<0.05, **P<0.01, ***P<0.001. For more details see Supplementary Figs 19 and 20.

To verify a causative role of PLA2g6 in impairment of the store-operated Ca^2+^ signalling and autophagic dysfunction, and to further link it to human PD, two molecular approaches were used. First, molecular rescue experiments were performed in ex2^KO^ MEFs (Fig. 6a–g) to determine if expression of WT PLA2g6 could recover normal store-operated Ca^2+^ signalling and autophagic function. Second, we tested if expression of a PLA2g6 mutant that is associated with human fPD could impair normal function of WT cells (Fig. 6h–n).

We found that deficient SOCE and depleted Ca^2+^ stores in ex2^KO^ cells can be effectively rescued by simple expression of the functional PLA2g6(L) (Fig. 6a,d). Restoration of SOCE and Ca^2+^ stores by WT PLA2g(L) was also sufficient to rescue autophagic flow and restore processing of LC3^mCherry–eGFP^ (Fig. 6e,g and Supplementary Fig. 19). Remarkably, expression of PLA2g6(L) that carries human fPD mutation^6^ (F72L, located in the N terminus of PLA2g6) failed to recover SOCE, or refill Ca^2+^ in the stores, and did not restore autophagic function (Fig. 6a–g and Supplementary Fig. 19). In contrast, the A80T mutant, which is not associated with human PD was able to fully restore normal Ca^2+^ signalling function (Fig. 6b,d).

To test if human PD-associated mutations in PLA2g6/*PARK14* indeed may cause targeted impairment of the store-operated Ca^2+^ signalling leading to autophagic dysfunction, the effects of acute expression of F72L and R747W mutants of PLA2g6(L) were tested in WT MEFs. Figure 6k–n demonstrate that these PD-associated mutants can indeed cause impairment of SOCE, depletion of the stores and impairment of autophagic flux. Importantly, the pathological effects of human PD-associated mutants were identical to those produced by expression of the N-terminally truncated PLA2g6(L) that mimics ex2^KO^ deficiency in our mouse model: expression of ex2^KO^ protein effectively impaired SOCE (Fig. 6k,l), depleted Ca^2+^ stores (Fig. 6m,n) and lead to significant autophagic dysfunction (Fig. 6h–j and Supplementary Fig. 20). The effects caused by the overexpression of these mutants were similar to what we found in MEFs from ex2^KO^ mice (Figs 2d–g and 4d,e), and in fibroblasts from the fPD^R747W^ patient. Thus, targeted impairment or restoration of PLA2g6 protein indeed can, respectively, impair or rescue store-operated Ca^2+^ signalling and autophagic function, further demonstrating direct association and leading role of PLA2g6 in these cellular events.

### PLA2g6(L) can rescue SOCE and autophagy in idPD cells

The results of the genetic and molecular manipulations with PLA2g6 validated a causal link between PLA2g6 deficiency, store-operated Ca^2+^ signalling and autophagy, and demonstrated that two PLA2g6(L) mutations associated with human fPD can cause impairment in SOCE, depletion of the stores and autophagic dysfunction. To determine if a similar sequence of pathological events could be associated with idPD, live-cell imaging and molecular rescue experiments were performed in primary human skin fibroblasts from idPD and control patients.

**Figure 7:**
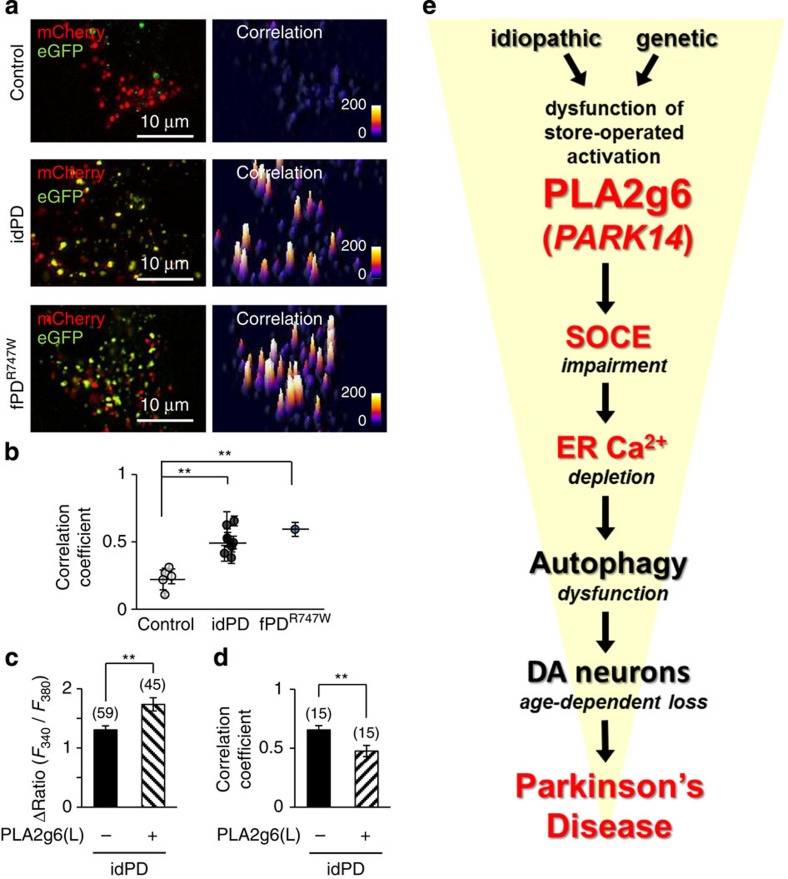
PLA2g6 (*PARK14*)-dependent Ca^2+^ signalling as a novel determinant of PD. (**a**) Representative images and correlation maps of LC3^mCherry–eGFP^ in live primary human skin fibroblasts from control, idPD and fPD PLA2g6^R747W^ mutant patients. (**b**) Summary data (average±s.e.) from experiments like in **a** show significant impairment of autophagy in idPD (*n*=10) and fPD^R747W^ (*n*=1) versus control (*n*=5) patients, which is evident from the higher correlation coefficient for LC3^mCherry–eGFP^ (see Supplementary Fig. 21 for details); **P<0.01. Results of the rescue experiments in live fibroblasts from idPD patient show (**c**) SOCE (like in experiments in Fig. 1a) and (**d**) correlation coefficient (like in experiments in Fig. 6a,b) 48 h after cells transfection with either PLA2g6(L) or empty vector as a control. Summary data show average±s.e. from the numbers of cells specified above the bars. **P<0.01. (**e**) Schematic illustration of a previously unknown sequence of pathological events that can be initiated by idiopathic or genetic deficiency in store-operated activation of PLA2g6 (*PARK14*), which can lead to SOCE impairment, depletion of intracellular Ca^2+^ stores and autophagic dysfunction, which results in progressive loss of DA neurons in SNc and age-dependent PD.

Analysis of primary skin fibroblasts from idPD and fPD^R747W^ patients revealed that in addition to reduced SOCE and depleted stores (Fig. 1), they all have significant autophagic dysfunction, as demonstrated by significant impairment of LC3^mCherry–eGFP^ flow (Fig. 7a,b and Supplementary Fig. 21). Thus, impairment of SOCE, depletion of ER Ca^2+^ stores and autophagic dysfunction (Fig. 7a,b) represent a new distinct sequence of pathological cellular events that could be found not only in DA neurons and MEFs from PLA2g6 ex2^KO^ mice but also in primary skin fibroblasts from idPD and fPD(*PARK14*) patients.

If specific defects in PLA2g6(L) expression (Fig. 1f) and/or store-dependent activation (Fig. 1e) could indeed be a major cause of SOCE deficiency and autophagic dysfunction in idPD cells, one would expect that overexpression of functional WT PLA2g6(L) should rescue SOCE and improve autophagic flux in idPD cells. Remarkably, the results of rescue experiments presented in Fig. 7c,d demonstrate that this prediction is correct, and PLA2g6(L) expression can indeed significantly improve SOCE and autophagic flux in idPD cells.

Altogether, the results in primary cells from idPD and fPD^R747W^ patients confirmed the critical role of PLA2g6(L) in impaired SOCE and autophagic dysfunction, and validated previously unknown association of these events with human idPD and *PARK14* fPD.

## Discussion

On the basis of our findings, we propose that dysfunction of PLA2g6-dependent Ca^2+^ signalling could be a previously unknown mechanism contributing to the pathophysiology of human PD. Figure 7e illustrates a sequence of pathological events that could be initiated by dysfunction of the store-dependent activation of PLA2g6, which we found in idiopathic and genetically induced forms of PD. Defects in PLA2g6(L) activation by depleted stores can be due to PD-associated mutations (like in fPD^R747W^ patient) or reduced expression (like in idPD patients) or cleavage of the N terminus (mimicked in ex2^KO^ mice), all of which were found to cause impairment of SOCE and depletion of ER Ca^2+^. It is tempting to speculate that the limited SOCE and low capacity of Ca^2+^ stores (Fig. 3) could make DA neurons particularly vulnerable to additional loss of SOCE found in idPD humans and PLA2g6 ex2^KO^ mice, thus setting the stage for their premature demise, which could be aggravated and accelerated by other factors that will be discussed below. Further, our studies present the unexpected discovery of a previously unknown ability of the defects in PLA2g6-dependent store-operated Ca^2+^ signalling to trigger autophagic dysfunction, premature loss of DA neurons in SNc and age-dependent Parkinsonism. This sequence of pathological events was validated in a novel PLA2g6 ex2^KO^ mouse model, which mimics deficient PLA2g6-dependent Ca^2+^ signalling and autophagic dysfunction found in idPD and fPD^R747W^ patients. Importantly, such defects in ex2^KO^ mice were directly associated with a marked age-dependent phenotype, which exhibits selective loss of DA neurons in the SNc and L-DOPA-sensitive motor dysfunction resembling human PD. The anatomic selectivity of age-dependent neurodegeneration in this model appears to be the best yet observed for PD-associated genetic defects in murine models^55^,^56^.

Discovery of a novel causal relationship between impaired SOCE, depleted stores, autophagic dysfunction and PD-like pathology illuminates complex role of Ca^2+^ signalling in PD. It creates an interesting paradigm: insufficient Ca^2+^ entry through SOCE mechanism can be as detrimental to DA neurons as excessive Ca^2+^ entry through voltage-gated Ca_V_1.3 channels. This apparent duality has a simple explanation: distinct Ca^2+^ entry pathways regulate different cellular processes^57^, and apparently can be linked to different pathological hallmarks of PD. Pioneering studies from Surmeier's laboratory showed that excessive Ca^2+^ entry through Ca_V_1.3 channels is linked to mitochondrial oxidant stress^58^,^59^, so that the physiological pacemaker activity of this Ca^2+^ channel increases pathological vulnerability of DA neurons. In contrast, Ca^2+^ entry through PLA2g6-independent^36^ TRPC1 channels was recently shown^60^ to be protective in a MPP+ (1-methyl-4-phenylpyridinium) model of DA neuronal cell death. Our current findings demonstrate that impairment of Ca^2+^ influx through the PLA2g6-dependent SOCE mechanism leads to depletion of ER Ca^2+^ stores, autophagic dysfunction and premature death of DA neurons, suggesting that preservation of SOCE and refilling of ER Ca^2+^ stores is essential for DA neuronal health and survival.

Why deficiency in SOCE, depleted ER Ca^2+^ stores and autophagic dysfunction may be particularly detrimental to DA neurons? Several reasons can be of particular importance, starting with the new empirical data demonstrating rather limited SOCE and low capacity of ER Ca stores in live iPSC-derived A9 midbrain DA neurons. Poor store-dependent Ca^2+^ signalling can increase vulnerability of DA neurons to mitochondrial stress imposed by constant pacemaker activity of Ca_V_1.3 channels (highlighted by the work of Surmeier's group^58^,^59^). This stress can be further exacerbated by idiopathic or genetic defect in PLA2g6-dependent Ca^2+^ signalling and autophagic dysfunction (found in idPD and fPD^R747W^ patients, and demonstrated in DA neurons from PLA2g6-deficient mice), which can push DA neurons over the threshold. Moreover, high demand for dopamine production makes DA neurons especially vulnerable to ER Ca^2+^ store depletion and autophagic dysfunction, which altogether may explain premature age-dependent demise of the nigral DA neurons in idPD patients and PLA2g6 ex2^KO^ mice.

Interestingly, distinct defects in Ca^2+^ signalling were recently associated with Alzheimer's disease (AD) and Huntington's (HD) disease: in contrast to PD, ER Ca^2+^ stores in AD and HD were found to be overfilled^61^,^62^. Moreover, Bezprozvany and colleagues reported that HD can be associated with TRPC1 overexpression and upregulation of TRPC1-dependent Ca^2+^ entry^62^. In contrast, AD can be associated with a significant reduction in synaptic expression of STIM2 leading to impairment of highly localized STIM2-dependent Ca^2+^ entry in mushroom spines and resulting in their loss^61^. Altogether, these findings suggest a rather high specificity of distinct Ca^2+^ signalling pathways for specific forms of neurodegeneration: pathological changes in expression or function of *PLA2g6*, *Orai1*, *TRPC1*, *STIM1*, *STIM2* or other molecules can affect distinct mechanisms of Ca^2+^ entry and/or storage, and lead to pathological changes in neurons (or distinct neuronal structures) that are most vulnerable to each specific defect.

The results of genetic and molecular manipulations with distinct molecules involved in SOCE presented new clues for the causality of PLA2g6(L)-dependent Ca^2+^ signalling events leading to autophagic dysfunction. We found that genetic ablation of Orai1 (in MEFs from Orai1^KO^ mice) mimics impairment of SOCE, ER store depletion and autophagic dysfunction found in PLA2g6 ex2^KO^ MEFs. Similar autophagic dysfunction can be also produced by TG, which inhibits SERCA and depletes ER Ca^2+^ stores. However, in contrast to TG, which activates SOCE, there is a significant loss of SOCE in Orai1^KO^ and PLA2g6 ex2^KO^ cells that have normal cytosolic Ca^2+^. Thus, PLA2g6-dependent depletion of Ca^2+^ stores (rather than actual reduction in SOCE) is most likely to be responsible for autophagic dysfunction, and loss of DA neurons in SNc leading to PD, as illustrated by the sequence of pathological processes proposed in Fig. 7e.

Impairment of the store-operated Ca^2+^ signalling might trigger or accelerate other pathological processes that are detrimental to DA neurons beyond the impairment of autophagy. For example, significant depletion of ER Ca^2+^ stores can also cause ER stress^63^ and unfolded protein response that plays important role in neurodegeneration^64^,^65^,^66^. It is important to emphasize that since PLA2g6-dependent Ca^2+^ signalling appears to be upstream of both autophagy and the unfolded protein response, its deficiency in DA neurons can contribute to (or set the stage for) aggregation to human α-synuclein and Lewy body formation, which is a diagnostic hallmark of PD^47^,^50^,^51^,^64^,^67^,^68^, and can be found in patients with familial *PARK14* mutations^7^. The late life onset of idPD and the late onset of PD-like phenotype in PLA2g6 ex2^KO^ mouse model indicate that additional age-dependent processes participate in the final demise of DA neurons that are put at risk by insufficient store-operated Ca^2+^ signalling (Fig. 3). Oxidative stress, mitochondrial dysfunction and/or protein misfolding are the hallmarks of a normal ageing process^44^, and while they do not by themselves cause PD in ageing WT mice, they may become lethal for DA neurons weakened by PLA2g6 deficiency, sustained Ca^2+^ store depletion and autophagic dysfunction. It is appealing to speculate that idiopathic or genetic loss of PLA2g6-dependent SOCE function could be especially detrimental to DA neurons, which are physiologically weakened by excessive mitochondrial oxidant stress due to the life-long pacemaker activity of Ca_V_1.3 channels. Our study supports the idea of nigrostriatal degenerative processes as a complex phenomenon^43^,^56^,^69^ that goes beyond mitochondrial dysfunction, oxidative damage and/or defects in protein degradation. It is likely that impairment of PLA2g6-dependent store-operated Ca^2+^ signalling can initiate or in tandem with other age-related processes exacerbate a sequence of pathological events leading to demise of specific DA neurons in SNc, and resulting in PD.

Altogether, our study presents empirical evidence for a previously unknown link between *PARK14*/PLA2g6, SOCE and human PD, and the data provide strong support for our conclusions. First, we found that primary skin fibroblasts from idPD patients have a characteristic cellular phenotype with pronounced deficiency in SOCE and significant autophagic dysfunction (Figs 1 and 7). Importantly, we demonstrated that such idPD phenotype (impaired SOCE and autophagic dysfunction) can be rescued by simple overexpression of the functional PLA2g6(L) (Fig. 7c,d). Second, the cells from fPD patient carrying PLA2g6^R747W^ mutation have the same cellular phenotype (loss of PLA2g6 activation by the stores, deficient SOCE, depleted stores and autophagic dysfunction) as we found in idPD patients (Figs 1 and 7). Third, human PD-associated mutations in *PARK14* (F72L and R747W) appeared to be sufficient to impair SOCE, deplete the stores and cause autophagic dysfunction (Fig. 6). Fourth, cellular phenotype of human idPD and *PARK14* fPD patients can be mimicked by targeted impairment of PLA2g6 function in a new transgenic mouse model (Figs 1, 2 and 6). Importantly, impairment of SOCE and depletion of Ca^2+^ stores was found not only in fibroblasts but also in iPSC-derived A9 midbrain DA neurons from PLA2g6 ex2^KO^ mice. Fifth, genetic impairment of Ca^2+^ signalling function of PLA2g6 in a new ex2^KO^ mouse model resulted in pronounced age-dependent PD-like phenotype that mimics idPD in humans.

The PLA2g6 ex2^KO^ mouse model exhibits not only PD-like motor dysfunction but also an anatomically selective depletion of nigral DA neurons resulting from an endogenous pan-neuronal deficit, and provides an important addition to existing mouse models of PD^55^,^56^. PLA2g6 ex2^KO^ mouse reproduces several major parameters of human sporadic PD, and combines autophagic dysfunction, progressive loss of DA neurons in SNc and age-dependent L-DOPA-sensitive PD-like motor dysfunction. This new mammalian model opens unique opportunities to investigate mechanisms contributing to the aetiology of sporadic PD, and provides a powerful tool for developing novel strategies for prevention and treatment of this devastating neurodegenerative disease.

## Methods

### Animals

A novel PLA2g6 ex2^KO^ transgenic mouse model, in which exon 2 of *Pla2g6* gene was constitutively deleted, was custom created by GenOway (http://www.genoway.com/, France). The strategy for creation and results of model validation are presented in Supplementary Methods and Supplementary Figs 5–7. Heterozygote ex2^KO^ males were backcrossed to C57BL/6 females for nine generations, and congenic B6.Cg-*Pla2g6*^*ΔEx2-VB*^/J line was established. Owing to infertility of homozygous ex2^KO^ males, and the inability of homozygous females to sustain neonatal pups, cross-breeding of heterozygous mice was employed to obtain homozygous ex2^KO^ animals used in this study. Ageing male mice were used for *in vivo* studies, while female mice were used for preparation of MEFs. Experimental sets of homozygous ex2^KO^ and WT littermate males were housed and aged together. Animal number for each study group was determined for the experimental results to reach statistical significance with a power of 90% at *P*<0.05, or to demonstrate that there is no difference between the groups. Animals were maintained in an advanced pathogen-free facility with veterinary service and unlimited access to food and water. All experimental procedures were compliant with ethical regulations and approved by the Institutional Animal Care and Use Committee of Boston University.

### Motor coordination tests

Ageing PLA2g6 ex2^KO^ and WT mice were monitored for the signs and severity of clinical symptoms, and motor deficit was initially assessed in arbitrary units (a.u.) using the following scale: 0=no abnormalities; 1=subtle signs of motor dysfunction; 2=clear signs of movement impairment, but sustained postural stability; 3=impairment in movement and occasional postural instability; 4=strong ataxia and instability, but no difficulty with eating, drinking and grooming; 5=very strong ataxia resulting in difficulty with keeping sternal/upright position, and frequently falls when walking, but still able to eat, drink and groom, although with some difficulty.

Analysis of motor function was performed in age-matched ex2^KO^ and WT animals using standard behaviour tests, as described in detail in Supplementary Methods. Balance beam test assessed the ability of ageing mice to maintain balance while walking along a narrow beam (the number of missteps per metre was counted). The pole test assessed the time that is needed for balance and orientation on the top of the pole. The rotarod test determined how long the mouse can maintain its balance and stay on a rotating rod. The grip test was used to objectively quantify the muscular strength of the forelimbs and hindlimbs. L-DOPA challenge test was performed on ex2^KO^ mice with motor deficits to determine the ability of L-DOPA to temporarily improve motor function, which was assessed using balance beam test. The data were summarized for each group as mean±s.e. The number of animals used for each study is identified on the graphs.

### Brain slices: preparation, immunostaining and analysis

The brains were extracted following paraformaldehyde (4%) perfusion, and cryopreserved in 15 and 30% (w/v) sucrose solutions at 4 °C. Brain sections were prepared using standard methods, as described in Supplementary Methods. Briefly, coronal 30-μm-thick free-floating sections containing the SNc were collected using a staggering method, and sets of six brain sections were collated: each set contained similar sections from the rostral, middle and caudal parts of the SNc region. Investigators were blinded during sectioning, TH staining, unbiased stereology and analysis of PAS staining; mouse phenotypes were revealed/confirmed only after data were generated. No samples were excluded from analysis in targeted age groups.

Blinded unbiased stereological analysis of DA neurons in SNc of age-matched pairs of ex2^KO^ and WT littermates was performed using standard methodology described in Supplementary Methods. Briefly, the matching sets of brain slices were stained with TH rabbit polyclonal antibody (Calbiochem) and 3,3′-diaminobenzidine horseradish peroxidase (HRP) substrate (Vector Laboratories). To estimate the number of TH+ neurons, matching sets of six sections from SNc area of the brain of each experimental animal were analysed using a Nikon Eclipse E600 microscope and the Stereo-Investigator v11.01.2 software. The total number of TH+ cells in the SNc was estimated using the optical fractionator technique, as described in Supplementary Methods. The data were summarized for each group as average±s.e. Paired *t*-test was used for statistical analysis of the differences within the matching pairs of WT and ex2^KO^ littermates. The numbers of littermate pairs used for these studies is identified on the graphs.

PAS staining and analysis was performed using Sigma kit (#395B) and standard procedures, as described in Supplementary Methods. PAS-positive cells (stained rose to magenta with blue to black nuclei) were counted in SNc, hippocampus and M1/M2 motor cortex regions. Summary data show average number of PAS+ neurons per mm^2^ (±s.e.) from three pairs of age-matched WT and ex2^KO^ 16-month-old animals.

Immunostaining for TH and LC3 was done using primary chicken polyclonal anti-TH antibody (Abcam ab76442), primary rabbit polyclonal anti-LC3 antibody (Cell Signaling #2775), secondary goat anti-rabbit Alexa488 (Invitrogen A11034) and goat anti-chicken Alexa594 (Abcam AB150172) antibodies, as described in Supplementary Methods.

Nissl (cresyl violet) staining was performed using a standard procedure described in Supplementary Methods.

### Primary MEFs

MEFs were isolated from WT, ex2^KO^ and Orai1^KO^ embryos (14.5 days old). Ex2^KO^ embryos were obtained from homozygote ex2^KO^ females mated with heterozygote ex2^KO^ males. Orai1^KO^ embryos were obtained by the cross-breeding of heterozygous Orai1^KO^ mice. Each embryo was genotyped. Head, vertebral column, dorsal root ganglia and all internal organs were removed and discarded; the remaining embryonic tissue was manually dissociated and incubated in 0.25% trypsin (Gibco) for 15–30 min. Cells from each embryo were plated onto a 10-cm tissue culture dish in MEF media (Dulbecco's modified Eagle medium (DMEM); Mediatech Inc.) containing 10% fetal bovine serum (FBS; Hyclone), non-essential amino acids, sodium pyruvate and penicillin/streptomycin (Invitrogen). After reaching confluence, primary MEFs from WT and ex2^KO^ embryos were tested (and confirmed to be negative) for mycoplasma contamination, collected and stored in liquid nitrogen for future use. Only MEFs from passages 2–3 were used for experiments, and studied after 24–48 h in culture. For each experimental condition, independent experiments were performed on the cells from three different MEF preparations; the number of cells (or samples) for each condition is shown in the figures, and/or stated in figure legends.

Transfection of MEFs was performed using the Amaxa Nucleofector system (Lonza, Allendale, NJ, USA). Briefly, the cells were plated in a six-well plate at a density of 200,000 per well. After 24 h the cells from each well were collected, centrifuged, resuspended in 100 μl of electroporation solution (Mirus Bio, Madison, WI, USA), mixed with 2 μg of the recombinant plasmid DNA and transfected using the T020 programme. After electroporation, the cells were plated in 2 ml of warm DMEM containing 10% FBS and 1% penicillin/streptomycin, and grown for 24–48 h, as specified. Transfection efficiency (verified by expression of GFP or LC3^mCherry–eGFP^) was >70%.

### iPSC-derived A9 midbrain DA neurons

Derivation of iPSC from MEFs was performed using STEMCCA approach (see Supplementary Methods for details). Colonies positive for alkaline phosphatase, Oct3/4, Zfp96, Nanog and ERas were expanded and banked for neural differentiation. The cultures were routinely checked and confirmed to be negative for mycoplasma.

Differentiation of iPSC into DA neurons was done using standard protocol^42^ by first inducing formation of embryoid bodies in non-adherent conditions for 4 days in KSR media. Cells were then transferred to adherent plates and incubated in ITS media (DMEM/F12 (Gibco)+1 × ITS Supplement (Sigma I13146)+1 μg ml^−1^ bovine fibronectin (Sigma F1141)) for 6–10 days to induce ectoderm formation. Cells were then expanded onto polyornithine-/fibronectin-coated coverslips in media containing N2 Max supplement, FGF2, FGF-8b, Shh-N and ascorbic acid for 5–7 days until cells reach confluency. Neural identity was confirmed by staining against βIII tubulin and nestin. Final differentiation into DA neurons was done by incubating the cells for 10 more days in minimal media (DMEM/F12 (Gibco)+1 × N2 Max+200 μM ascorbic acid (Sigma A4403)). Confirmation of DA neuron identity was done by staining the cells for TH (Abcam ab76442), Dopamine transporter (Abcam ab5990) and vesicular monoamine transporter 2 (Abcam 70808).

### Human primary skin fibroblasts

The samples of hPSF (see Supplementary Table 1 for all details) were obtained from the NINDS Cell Line Repository (http://ccr.coriell.org/ninds). Each sample was verified to be mycoplasma-free by PCR. The hPSFs from individual donors were cultured as recommended. Briefly, hPSF cells were grown in Eagle's minimum essential medium (Gibco) supplemented with 15% FBS (Atlanta Biologicals, GA, USA), 2 mM L-glutamine (Gibco) and 1% penicillin/streptomycin (Gibco), and passaged 1:4 every 7 days. Only hPSF from early passages (3–5) were used in these studies. For each experimental condition, at least three independent experiments were performed; the numbers of cells (or samples) for each condition is shown in the figures, and/or stated in figure legends.

Transfection of hPSFs was performed using the Amaxa Nucleofector system. The cells were plated at a density of 300,000 per well in a six-well plate. After 24 h the cells from individual wells were collected, centrifuged, resuspended in 100 μl of electroporation solution, mixed with 2 μg of total plasmid DNA and transfected using the U023 programme. After electroporation, the cells were plated in fibronectin-coated (2.5 μg cm^−^^2^, Sigma, St Louis, MO, USA) 35-mm glass-bottom dishes. Live-cell imaging was performed 48 h after transfection. Transfection efficiency (verified by expression of GFP or LC3^mCherry–eGFP^) was about 50%.

### Ca^2+^ imaging

Intracellular Ca^2+^ studies were performed using standard Fura-2 imaging technique (for details see Supplementary Methods). Briefly, the cells were loaded with Fura-2/AM (5 μM; Invitrogen) and cytosolic Ca^2+^ (using *F*_340_/*F*_380_ ratio) was recorded simultaneously in individual cells. Representative traces in the figures show average (±s.d.) responses from up to 20 cells recorded simultaneously. Ca^2+^ changes are shown by ΔRatio (Δ*F*_340_/*F*_380_), which is the difference between the basal and peak values of Ca^2+^ responses. Summary bar graphs show the average (±s.e.) from three independent experiments for each condition; the total number of cells in each experimental group is identified on the graphs.

For SOCE recording, the cells were placed in Ca^2+^-free extracellular solution (130 mM NaCl, 4.6 mM KCl, 2 mM MgCl_2_, 10 mM HEPES/Na, 5 mM glucose, 100 μM EGTA, pH 7.4), and acute (5–20 min) application of 5 μM TG (Sigma) was used to irreversibly inhibit SERCA activity, and allow Ca^2+^ to leak out from the stores, thus causing ER store depletion. Acute treatment with TG did not cause unfolded protein response (Supplementary Fig. 4). SOCE was measured in response to extracellular application of 2–2.5 mM Ca^2+^ in the presence of TG. Concentration and time of acute TG treatment was titrated for each cell type to ensure >90% loss of Ca^2+^ from TG-sensitive stores at the time of Ca^2+^ addition. As an alternative to TG, in some experiments SOCE was evoked by acute 5-min treatment with 400 μM TPEN (*N*,*N*,*N′*,*N′*-Tetrakis(2-pyridylmethyl) ethylenediamine, Sigma, USA). To inhibit PLA2g6 activity, (S)-BEL (S-bromoenol lactone, Cayman, USA) was applied (10–50 μM for 20 min in serum-free medium at 37 °C) to the cells after their loading with Fura-2/AM, and was washed away before the start of experiment.

Release of Ca^2+^ from intracellular stores was measured in Ca^2+^-free extracellular solution in response to acute application of ionomycin (Sigma) at concentration enough to release >90% of Ca^2+^ from TG-sensitive stores (titrated for each cell type): 1 μM for MEFs, 0.1 μM for hPSF and 0.1 μM for iPSC-derived DA neurons.

Fura-2 recordings in MEFs were done at 20–22 °C using dual-excitation Intracellular Imaging system (Intracellular Imaging Inc., Cincinnati, OH), unless specified differently. Fura-2 fluorescence in hPSF and iPSC-derived DA neurons was recorded using Nikon Eclipse Ti (Nikon, Melville, NY)/Lambda DG-4 system (Sutter Instrument, Novato, CA) equipped with Fura-2 cube (FURA2-C-NTE-ZERO, Semrock, Rochester, NY), CFI S Plan Fluor × 20/0.45 objective (Nikon), perfect focus, X/Y positioning and multiple field stitching. Fura-2 recordings in iPSC-derived neurons were done at 37 °C and the data from the whole field, which were labelled with a scratch for later identification, were stored. The cells were then immediately fixed, stained for TH and imaged. The paired images (Fura-2 recordings and ICC stainings) were superimposed and Ca^2+^ responses were analysed in TH+ iPSC-derived A9 midbrain DA neurons.

For comparison of Ca^2+^ responses in iPSC-derived DA neurons and MEFs, a separate set of experiments was performed with both cell types treated and studied at exactly the same experimental conditions (37 °C) using the same settings on Nikon Eclipse Ti/Lambda DG-4 system.

### Live-cell imaging and co-localization analysis

Live-cell imaging was done using Nikon Ti inverted fluorescence microscope equipped with a Perfect Focus system and environmental chamber (InVivo Scientific). Live cells transfected with LC3^mCherry–eGFP^ (alone, or in combination with PLA2g6, or its mutants) were imaged in glass-bottom dishes in culture medium at 37 °C and 5% CO_2_. Images of individual cells were taken using a × 60/1.4 Plan-Apochromat oil immersion objective (Nikon) and filter sets for GFP (excitation: 465–496; emission: 515–555) and Texas Red (excitation: 540–580; emission: 600–660). Images of representative cells were analysed using ImageJ (Wayne Rasband, NIH) software. For increased accuracy and better visualization, the background was subtracted (rolling ball radius: 5.35 μm), and the unsharp mask filter (radius: 0.428 μm; mask weight: 0.6) was identically applied to both green and red channels for all images. Analysis of mCherry and eGFP co-localization was done using Pearson's correlation coefficient^70^. Briefly, the normalized covariance image of each cell was computed using ImageJ and the equation:


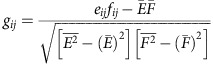


where *e*_*ij*_ and *f*_*ij*_ are the pixels in the respective panels, 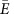 and 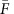 are the mean values of the pixels and 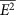 and 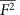 are the means of the squared pixel values. The average of the values of all pixels *g*_*ij*_ was equal to Pearson's correlation coefficient. A correlation map was generated using the Interactive 3D surface plot plugin in ImageJ, and the positive contribution at each pixel to the correlation coefficient is displayed. Summary data show the average±s.e. of correlation coefficients and sizes of fluorescent particles in a total of 15 cells from three independent experiments (five representative cells per experiment).

### Confocal and TIRF imaging

Confocal imaging of brain slices was done using LSM710 Duo imaging system (Zeiss, Thornwood, NY,USA) with either a × 20 non-immersion or × 63/1.4 Plan-Apochromat oil immersion objectives, as described in Supplementary Methods. Total internal reflection fluorescence (TIRF) imaging of the bottom plane of MEFs was done using a Nikon Ti inverted fluorescence microscope with a × 60/1.49 Apo-TIRF oil objective (Nikon) and a filter set for GFP illumination.

### PLA2g6 activity

A modified PLA2 assay kit (Cayman for MEFs and AbCam for hPSF) was used as previously described^21^,^26^. Briefly, each sample of live hPSF or MEF cells was homogenized using a cold lysis buffer (10 mM Tris-HCl, pH 7.0, 300 mM sucrose, 0.5% Triton X-100). To identify specific activity of Ca^2+^-independent PLA2g6, the assay buffers were modified to contain no Ca^2+^. To assess catalytic activity of PLA2g6, the cells were homogenized and treated with 10 mM EGTA for 10 min, which is known to directly displace inhibitory calmodulin and fully activate PLA2g6. For the analysis of PLA2g6 activation by store depletion, live cells were pretreated with TG (5 μM for 10 min) before homogenization, and homogenates were not treated with 10 mM EGTA. The specificity of PLA2g6 activity in both cases was confirmed by its inhibition with S-BEL(25 μM for 10 min), a chiral-specific suicidal substrate^38^ that discriminates PLA2g6 from all other phospholipases. PLA2g6 activity in each sample was assayed (in triplicates) by incubating the samples with the substrate, 1-hexadecyl-2-arachidonoylthio-2-deoxy-*sn*-glycero-3-phosphorylcholine for 1 h at room temperature in a modified Ca^2+^-free assay buffer (10 mM HEPES, pH 7.4, 300 mM NaCl, 60% glycerol, 8 mM Triton X-100, 4 mM EGTA and 2 mg ml^−1^ bovine serum albumin). The reaction was stopped and the generation of free thiols was visualized by addition of DTNB (5,5′-dithiobis-(2-nitrobenzoic acid)) for 5 min: the absorbance was determined at 405 nm using a standard microplate reader. In calculations of specific PLA2g6 activity a value of 10 mM^−1^ was used as extinction coefficient for DTNB at 405 nm.

The activity of PLA2g6 was expressed in nM min^−1^ per mg of protein.

### Western blot

Tissue preparation and western blot analysis was done using standard approaches (see Supplementary Methods for details).

### Antibodies

The following primary antibodies were used in this study: rabbit polyclonal anti-TH antibody (Calbiochem, #657012); chicken polyclonal anti-TH antibody (Abcam, ab76442); rabbit polyclonal anti-LC3B antibody (Cell Signaling, #2775); rabbit polyclonal anti-vesicular monoamine transporter 2 antibody (Abcam, ab70808); rat monoclonal anti-dopamine transporter antibody (Abcam, ab5990); rabbit polyclonal anti-PIN antibody targeting mouse PLA2g6 PIN domain (encoded by exon 8b, which is present in (L), but spliced out in (S) variant) were custom made by Yenzym Antibodies, LLC (San Francisco, CA), rabbit polyclonal anti-LC3B antibody (MBL, PD014); and monoclonal anti-β-actin antibody (Sigma, A1978). Secondary antibodies are the following: goat anti-rabbit Alexa488 (Invitrogen A11034), goat anti-chicken Alexa594 (Abcam ab150172) and goat anti-rat Alexa647 (Abcam ab150167) were used for imaging; HRP-conjugated anti-rabbit (Dako, K4002) was used for 3,3′-diaminobenzidine staining; HRP-conjugated anti-mouse (Cell Signaling, #7076) or anti-rabbit IgG (Cell Signaling, 7074) was used as secondary Ab for western blots.

### Quantitative reverse transcription–PCR

Total RNA was isolated from hPSF of each individual donor and from primary MEFs from ex2^KO^ and WT mice using High Pure RNA isolation kit (Roche Applied Science). Concentration and quality of samples were confirmed spectrophotometrically. RNA was reverse transcribed using High Capacity RNA-to-cDNA Kit (Life Technologies), and cDNA (equivalent of 200 ng RNA) was analysed per each reaction (in duplicates for technical control) in quantitative PCR on StepOnePlus Real Time PCR System (Applied Biosystems). For the full list of TaqMan gene expression assays, see Supplementary Methods. The relative expression level for each gene was normalized to the level of GAPDH in the same sample.

### DNA constructs

Tandem LC3^mCherry–cGFP^ construct was obtained from Addgene. cDNA for human PLA2g6(L) variant^39^ (Genbank #AF064594) was a kind gift from Dr Brian P. Kennedy (Karolinska Institute, Stockholm, Sweden) and MERCK FROSST CANADA Inc. His-tagged and/or myc-tagged expression constructs of PLA2g6(L) were created by PCR subcloning of the full-length long variant of human PLA2g6(L) into pcDNA3.1 plasmid (see Supplementary Methods for details). F72L and A80T mutants of ^myc^PLA2g6(L)^his^ were created by Mutagenex (USA), and confirmed by sequencing.

### Statistical analysis

A two-sided unpaired *t*-test was used for comparison among different data sets, unless stated differently. Normal distribution was confirmed using D'Agostino–Pearson omnibus normality test (*α*<0.05). A two-sided paired *t*-test was used for stereological analysis. One-way analysis of variance was used for the analysis of the data from L-DOPA challenge test. The difference between data sets was considered significant at *P*<0.05; *P* values are identified in the figures and legends as *P<0.05, **P<0.01, ***P<0.001. Summary data represent average±s.e. or s.d., as specified in the figure legends.

## Additional information

**How to cite this article:** Zhou, Q. *et al.* Impairment of *PARK14*-dependent Ca^2+^ signalling is a novel determinant of Parkinson's disease. *Nat. Commun.* 7:10332 doi: 10.1038/ncomms10332 (2016).

## Supplementary Material

Supplementary Information Supplementary Figures 1-22, Supplementary Table 1, Supplementary Methods and Supplementary References.
