# Supplementary material for: Impairment of PARK14-dependent Ca2+ signalling is a novel determinant of Parkinson's disease
Source: Nat Commun. 2016 Jan 12;7:10332. doi: 10.1038/ncomms10332 (PMC4729940; doi:10.1038/ncomms10332)
Supplement: Supplementary Information — Supplementary Figures 1-22, Supplementary Table 1, Supplementary Methods and Supplementary References. [file ncomms10332-s1.pdf]

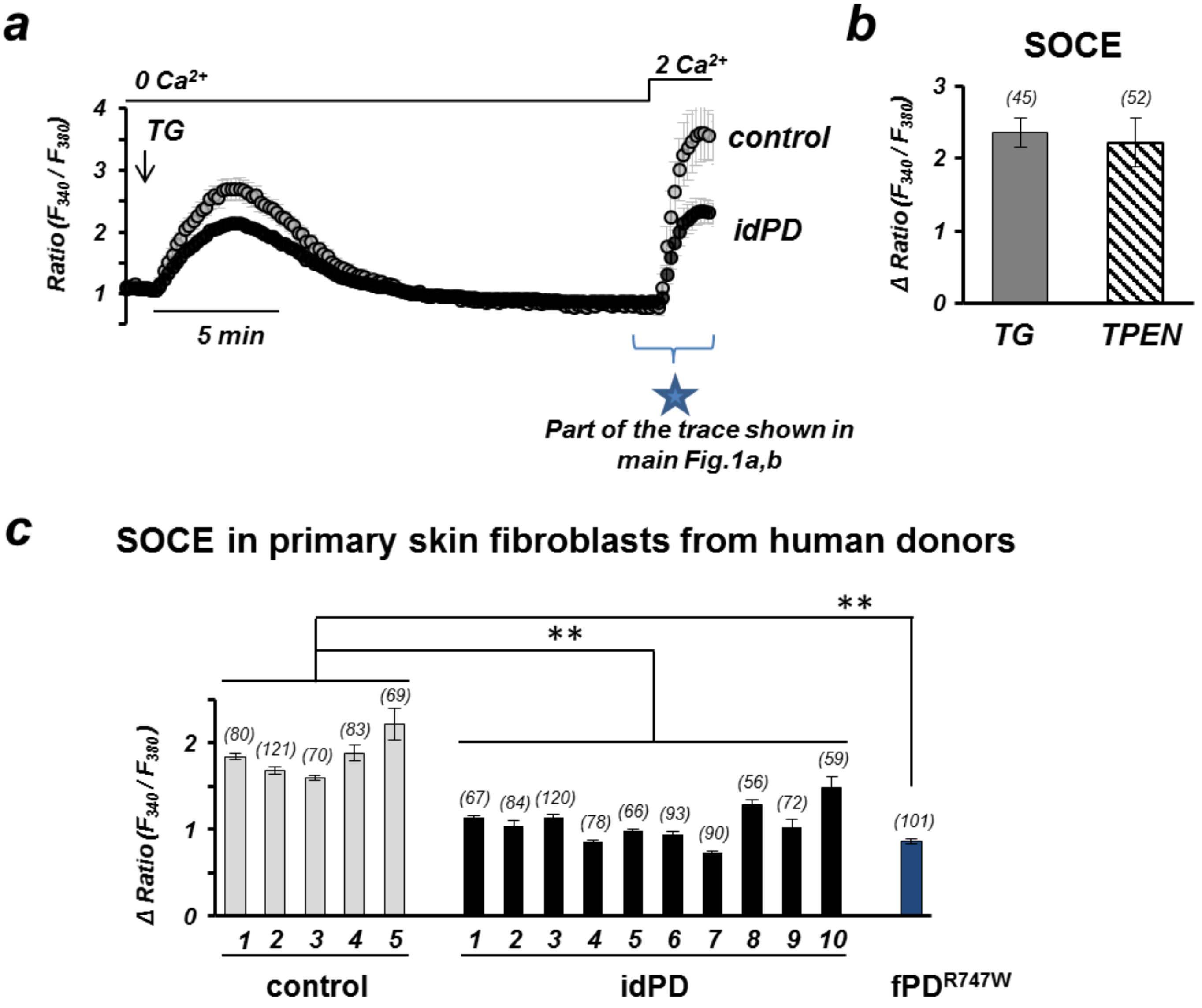

<u>**Supplementary Figure 1**</u>

Store-operated Ca^{2+} entry (SOCE) in primary skin fibroblasts (hPSF) from human donors.

(a) Representative trace showing changes in cytosolic Ca^{2+} following thapsigargin (TG, 5μM) application in the absence of extracellular Ca^{2+} (initial Ca^{2+} rise due to passive Ca^{2+} leak from ER stores), followed by SOCE (second Ca^{2+} rise due to Ca^{2+} influx into the cells upon extracellular Ca^{2+} addition). The average Fura2 Ratio (F_{340}/F_{380}) ±SD from a group of 10-20 individual cells measured simultaneously. <u>The blue star identifies the part of the experiment that is chosen for representative traces that illustrate SOCE in the Main Figure 1a,b.</u>

(b) Store-operated Ca^{2+} entry (SOCE) in hPSF can be triggered by either TG-induced inhibition of SERCA (that allows passive Ca^{2+} leak from the stores), OR by TPEN-induced buffering of free Ca^{2+} in ER stores.

(c) Comparative analysis of SOCE in primary skin fibroblasts from 5 control donors, 10 patients with idiopathic PD (idPD), and a patient with familial PD caused by R747W mutation in PLA2g6. Data for each patient show the average ±SE from at least 3 independent experiments, with up to 120 cells analyzed for each patient; ** $p<0.01$.

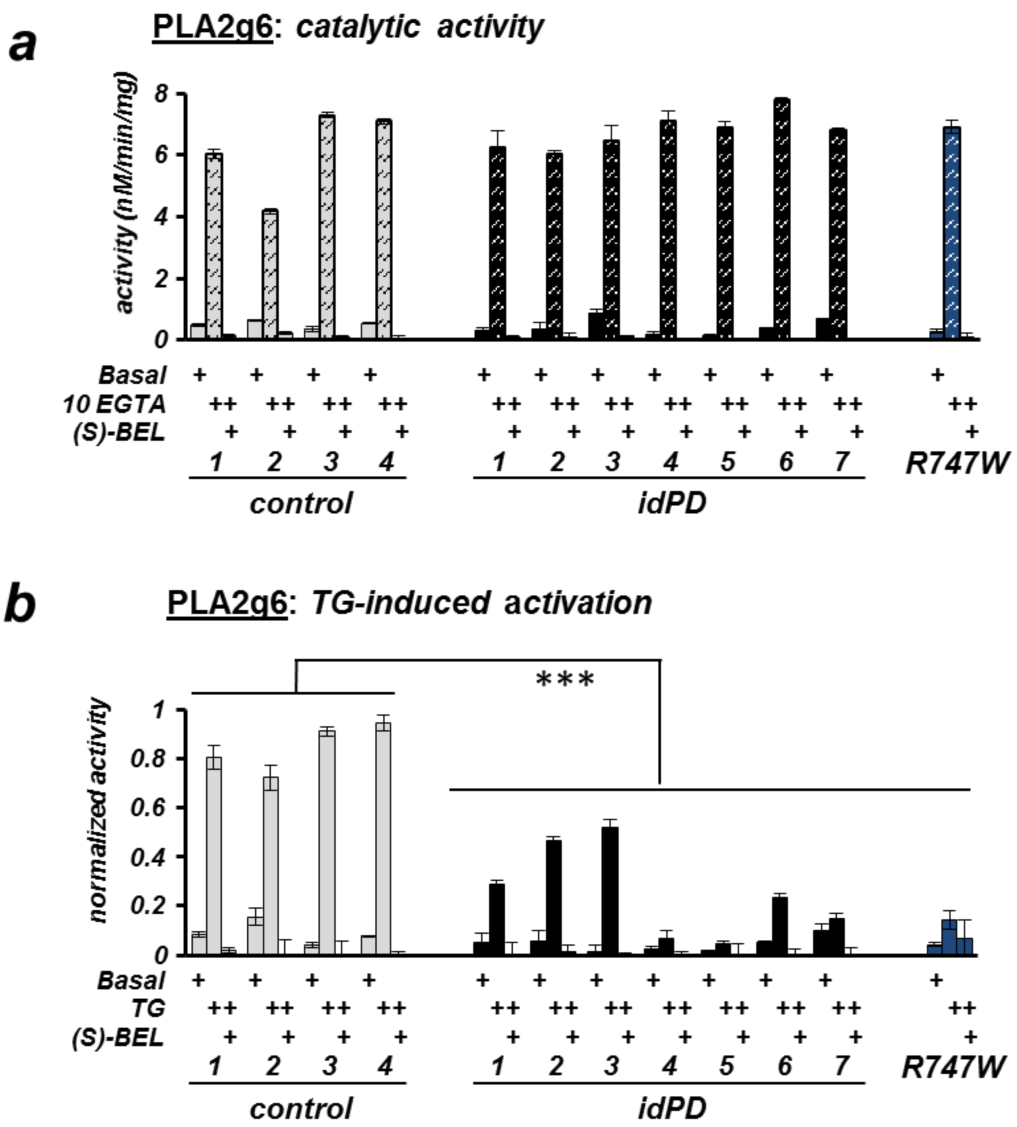

Supplementary Figure 2

Catalytic activity and TG-induced activation of PLA2g6 in primary skin fibroblasts (hPSF) from idiopathic PD (idPD) patients, familial PD patient with R747W mutation in PLA2g6, and control individuals.

For analysis of the catalytic activity of PLA2g6, the cells were homogenized and treated with 10mM EGTA, which is known to directly displace inhibitory calmodulin and fully activate PLA2g6. To assess store depletion-induced activation of PLA2g6, live cells were pretreated with TG before homogenization, and homogenates were not treated with 10mM EGTA. The specificity of PLA2g6 activity in both cases was confirmed by its inhibition with S-BEL, a chiral-specific suicidal substrate that discriminates PLA2g6 from all other phospholipases.

(a) Summary data show catalytic PLA2g6 activity in homogenates of hPSF from each individual donor: summary results for each patient show average activity (±SEM) from 3 repetitions under basal conditions, after PLA2g6 was fully activated by 10mM EGTA-induced displacement of inhibitory CaM, and after PLA2g6 inhibition with 25 μM of (S)-BEL.

(b) Summary data show activation of PLA2g6 by TG-induced depletion of Ca^{2+} stores in intact hPSF from individual control and idPD patients: summary data for each patient show average activity (±SEM) from 3 repetitions in basal conditions, after activation by TG (5 μM for 10 minutes), and after inhibition with (S)-BEL, normalized to catalytic activity for each patient. *** $p<0.001$.

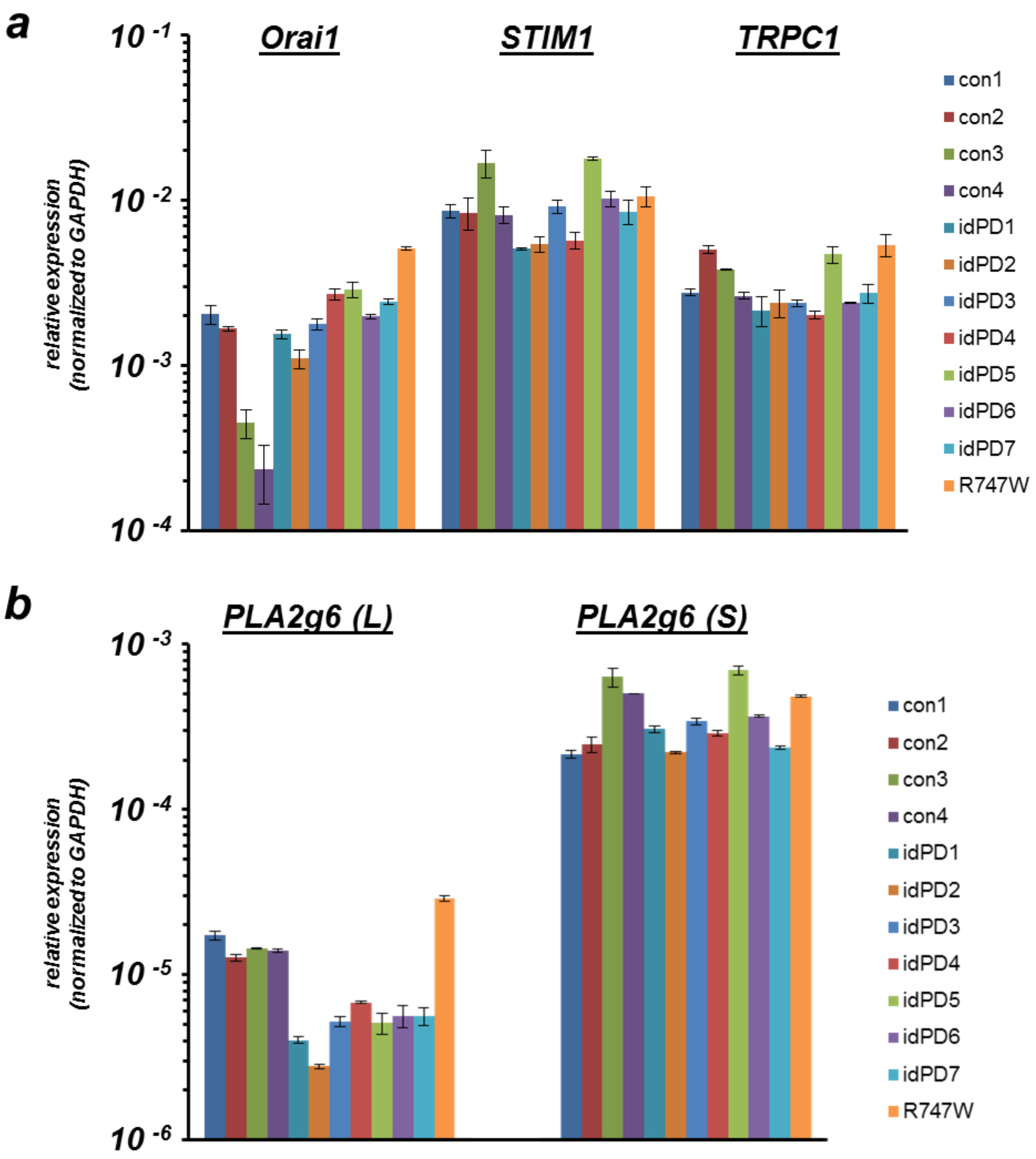

<u>Supplementary Figure 3</u>

Expression levels of Orai1, STIM1, TRPC1, and PLA2g6 in human primary skin fibroblasts (hPSF) from control individuals (con1-4), idiopathic PD (idPD) patients (idPD1-7), and familial PD patient with R747W mutation in PLA2g6 (PLA2g6^{R747W}).

Results of the quantitative Real Time PCR (qRT-PCR) analysis of expression levels of: ***(a)*** *Orai1, STIM1, TRPC1*, and ***(b)*** two major splice variants of *Pla2g6* gene: full length *PLA2g6(L)* and shorter *PLA2g6(S)* in which exon 8b is spliced out. Each data point is average ±SE (n=2) normalized to the level of *GAPDH* expression in corresponding sample from each patient.

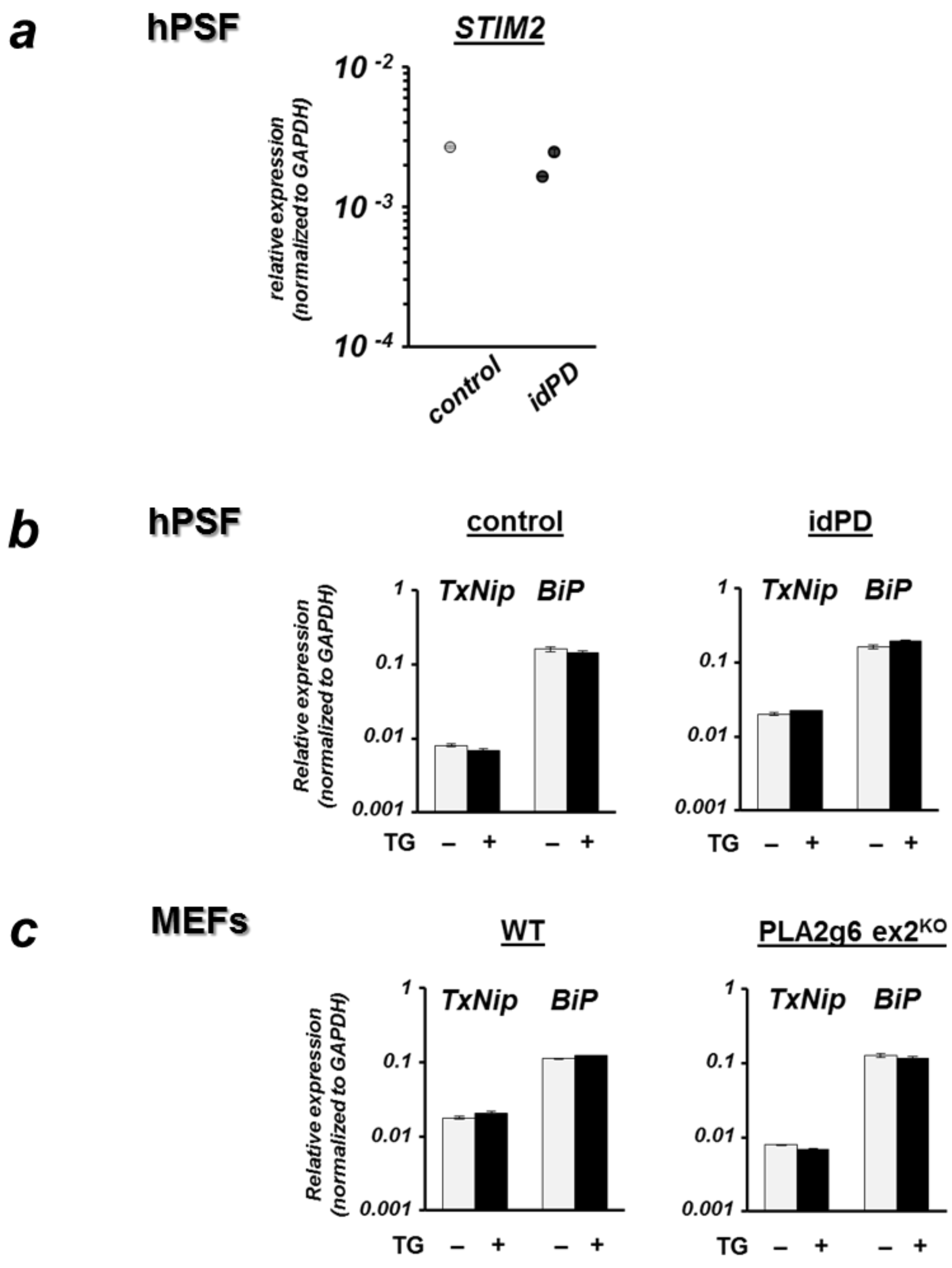

Supplementary Figure 4

Results of the quantitative Real Time PCR (qRT-PCR) analysis show:

(a) no difference in basal *STIM2* expression in hPSF from control and idPD patients,

(b) no effect of acute TG treatment (5μM, 20 min, like in Supplementary Fig. 1a) on *TxNip* and *BiP* expression in hPSF from control and idPD patients;

(c) no effect of acute TG treatment (5μM, 5 min, like in Main Fig. 2d) on *TxNip* and *BiP* expression in MEFs from WT and PLA2g6 ex2^{KO} mice.

Each data point is average ±SE (n=2) expression of each target normalized to the level of *GAPDH* expression in corresponding sample.

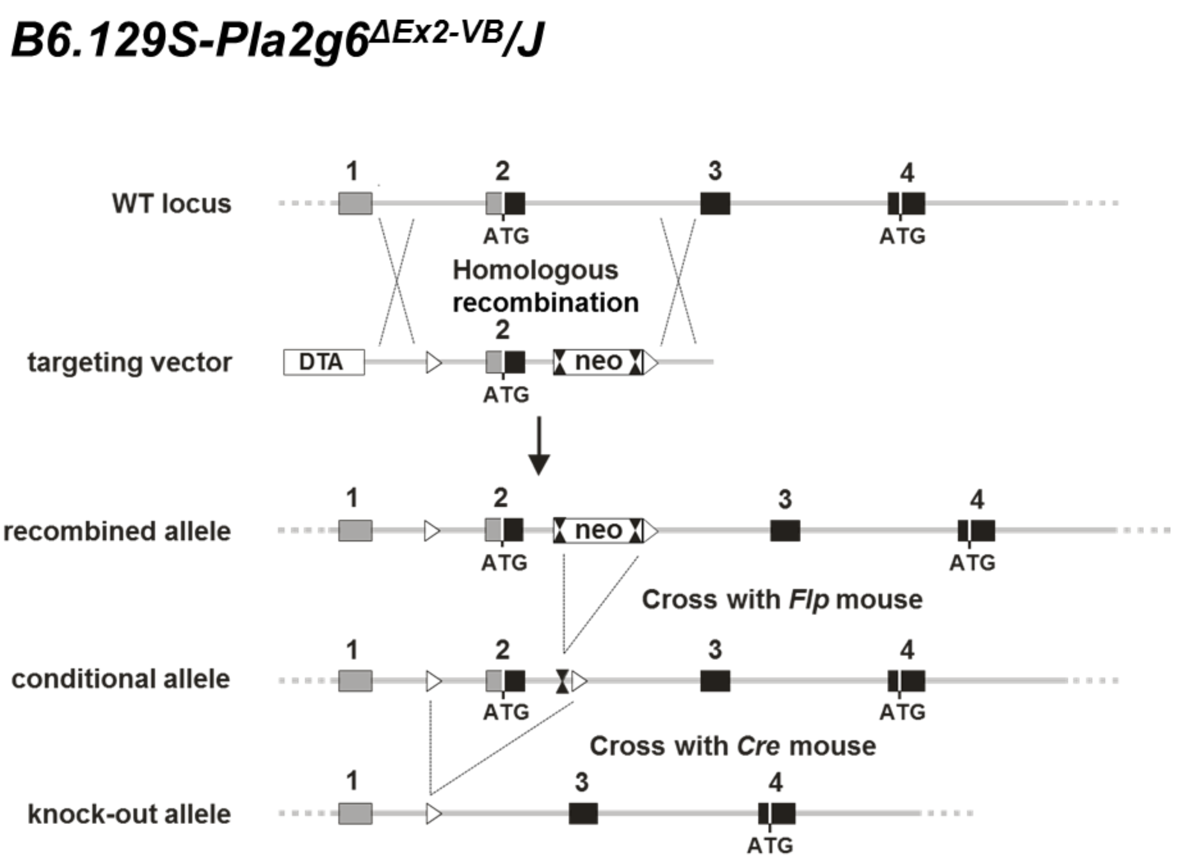

<u>**Supplementary Figure 5**</u>

Generation of constitutive PARK14 (PLA2g6) ex2^{KO} mouse model (B6.129S-Pla2g6$^{\Delta Ex2\text{-}VB}$/J).

WT *Pla2g6* locus and the targeting vector are schematically represented at the top of the panel. Exon 2 of *Pla2g6*, containing the translation initiation codon, is flanked by two *loxP* sites (open triangles), whereas the neomycin cassette (Neo) is immediately flanked by two *FRT* sites (double filled triangles). As depicted, the expected homologous recombination event creates the recombined (floxed) locus and removes Diphtheria Toxin A (DTA) negative selection marker. Crossing a recombined *Pla2g6* locus mouse with a ubiquitous Flp recombinase C57BL/6 animal allowed for excision of the FRT-flanked region, creating an animal carrying conditional *Pla2g6^{Ex2}* allele without neomycin selection cassette. Breeding the heterozygous recombined F1 mouse with a ubiquitous Cre recombinase C57BL/6 animal resulted in the Cre-mediated excision of the floxed exon 2 region, creating a total *exon 2* knockout (ex2^{KO}) mouse. For other details, see Supplementary Methods.

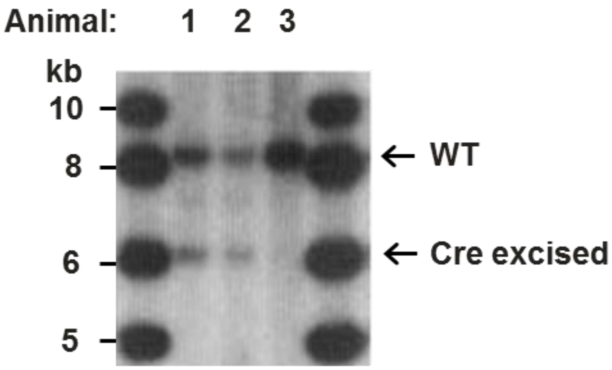

<u>**Supplementary Figure 6**</u>

Southern blot confirmation of the constitutive Pla2g6(ex2) knockout.

The genomic DNA of the 2 tested F1 mice (lanes 1 and 2) were compared with wild-type DNA (lane 3). The HpaI/NheI digested DNAs were blotted on nylon membrane and hybridized with the probe expected to anneal to the 3’ end of homology arm of the targeting vector to validate the zygocity of the *Pla2g6(ex2)* constitutive knock-out gene mutation in these animals. The expected fragments are: 8.2, 9, and 6.1 kb for WT allele, recombined/floxed allele, and constitutive knock-out allele (floxed region deleted), respectively.

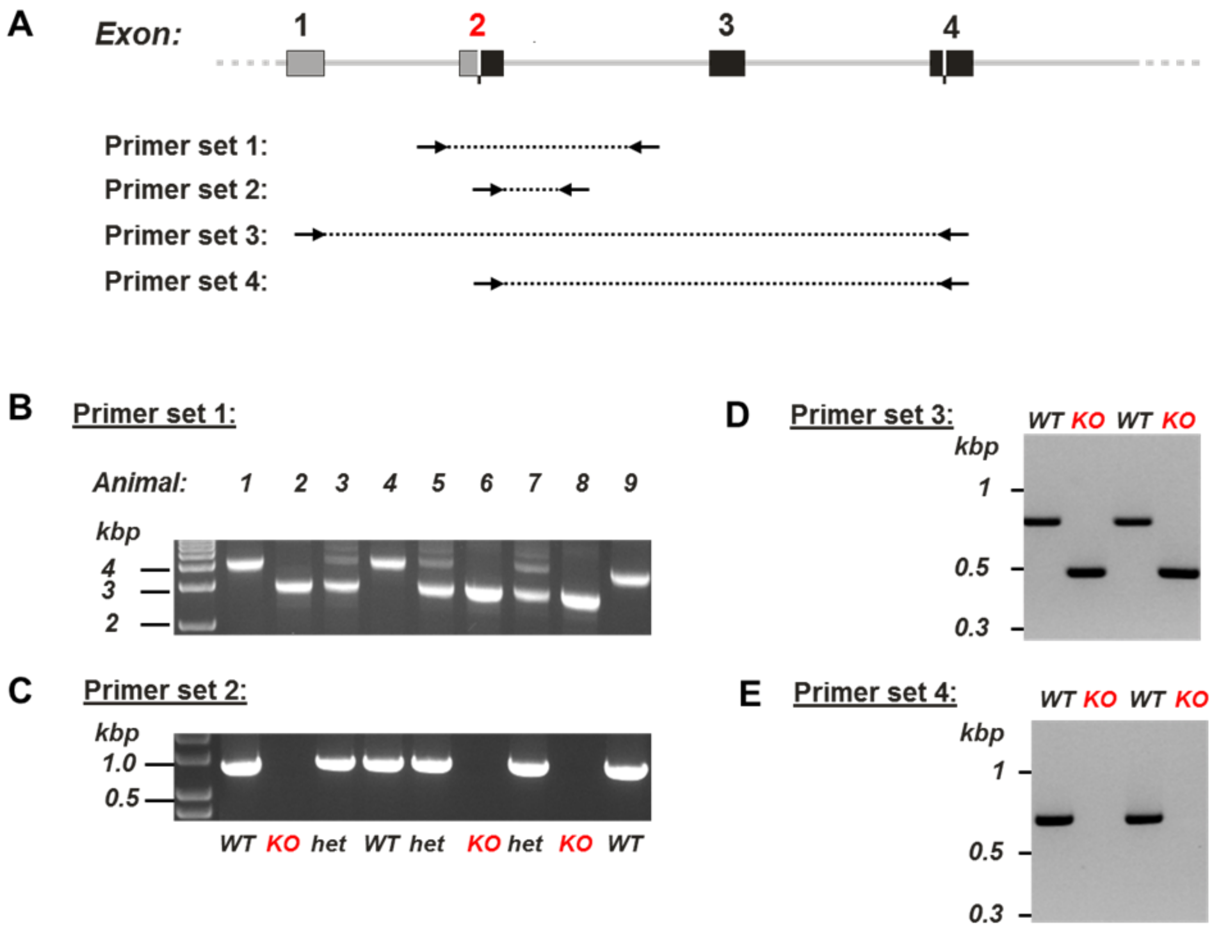

Supplementary Figure 7

PCR-based genotyping and confirmation of the constitutive Exon 2 knockout at the genome and transcript level.

(A) Schematic representation of WT *Pla2g6* locus with positions of four sets of primers used for PCR-based genotyping (sets 1 and 2) of the colony, or for confirmation of the lack of *Pla2g6* exon 2 in transcripts from mouse brains (set 3 and 4). (**B)** and **(C)**, Representative results of tail DNA genotyping for 9 animals from the colony using PCR primer sets 1 and 2. Expected length of PCR products for primer set 1 are 4028 (WT) and 2900 bp (ex2 KO allele), and for set 2 only WT allele (857 bp product) can be detected. Taken together, PCR with both sets of primers allowed for unambiguous determination of the *Pla2g6* locus genotype for each animal within the colony. **(D)** and **(E)**, Total RNA isolated from brains of two representative pairs of WT and exon 2 KO animals was reverse-transcribed and used as a template for PCR with primer sets 3 and 4. Expected length of PCR products for primer set 3 are 736 (WT) and 486 bp (ex2 KO allele), and for set 4 only WT allele (644 bp product) can be detected. As expected, for both animals previously genotyped as Exon 2 KO (using primer sets 1 and 2), transcripts coding for PLA2g6 are present in the brain, but are missing exon 2. Additionally, the product amplified with the primer set 3 from brains of ex2^{KO} mice was cloned and sequenced, and both the expected cDNA sequence and the lack of Exon 2 were confirmed (data not shown).

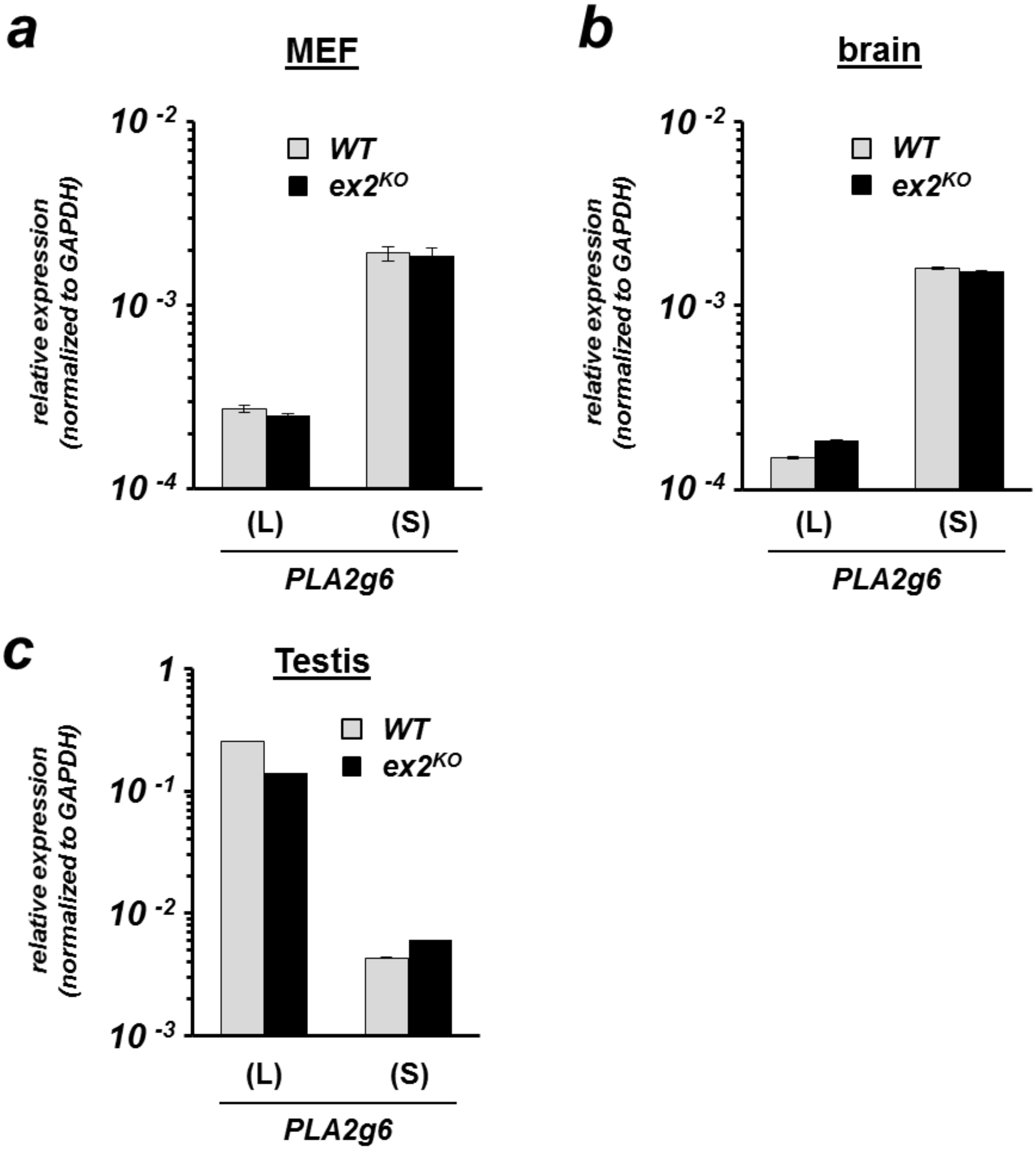

Supplementary Figure 8

Knock out of Exon 2 of Pla2g6 gene did not affect the level of transcripts of the (L) and (S) splice variants of PLA2g6 in the brain (a), MEF cells (b) and testis (c).

Quantitative Real Time PCR analysis of expression levels of (L) and (S) splice variants *of PLA2g6* in the brains, testis and MEF cells from WT and $ex2^{KO}$ mice. Please, notice significantly higher expression of *PLA2g6(L)* in testis. Data are normalized to *GAPDH* in each sample, and shown as average ±SE from 2-3 experiments.

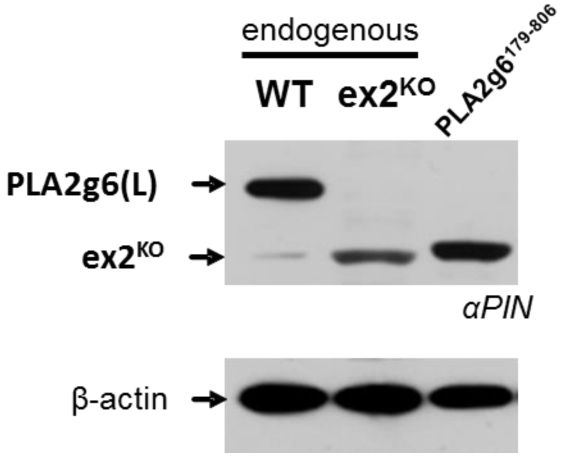

Supplementary Figure 9

Full length PLA2g6(L) is present in WT, while only truncated protein is present in ex2^{KO} mice.

Representative Western blot probed with custom-made PIN antibody that specifically targets PIN domain encoded by exon 8b that is present only in (L) splice variant of PLA2g6, and β-actin staining of the same samples. Specificity of PIN ab is shown in Supplementary Fig. 10. Images have been cropped for presentation. Full size images are presented in Supplementary Fig. 22.

WT: endogenous protein from testis of WT mouse;

ex2^{KO}: endogenous protein from testis of ex2^{KO} mouse;

PLA2g6$^{179\text{-}806}$: recombinant N terminally truncated myc/his-tagged PLA2g6(L)$^{179\text{-}806}$ protein expressed in FreeStyle™ 293-F cells. Please, notice that recombinant protein contains myc and his tags on its N and C termini, respectively, which slightly increase its MW in comparison with equivalent endogenous protein in ex2^{KO} mice.

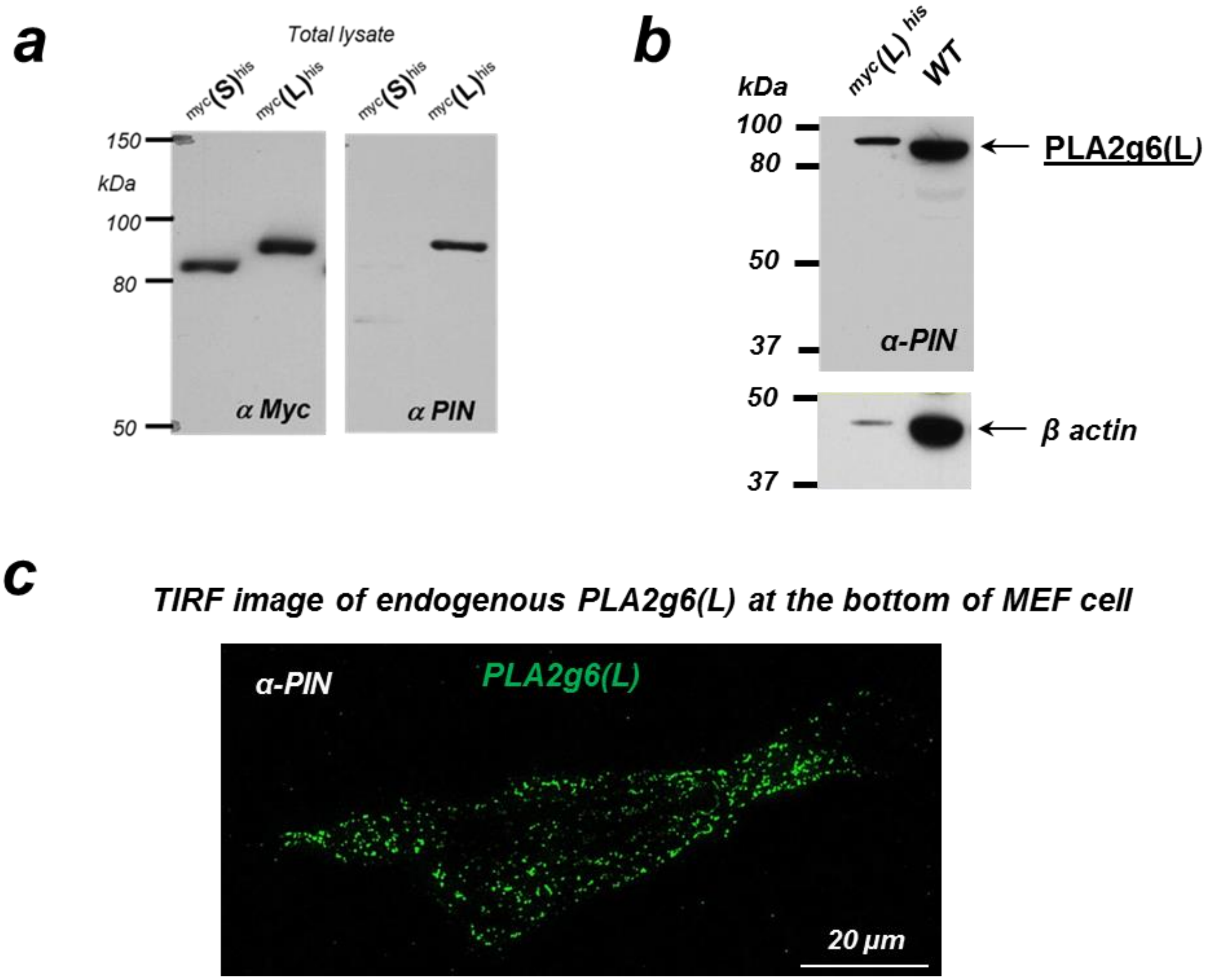

Supplementary Figure 10

Validation of custom mPIN ab that specifically recognizes (L), but not (S) variant of PLA2g6.

(a) Representative Western blot shows that recombinant ^{myc}PLA2g6(S)^{his} and ^{myc}PLA2g6(L)^{his} protein can be detected with Myc antibody, while only ^{myc}PLA2g6(L)^{his} protein can be recognized by custom-made PIN antibody that specifically targets PIN domain (encoded by exon 8b), which is present in (L), but spliced out in (S) variant of PLA2g6. Images have been cropped for presentation. Full size images are presented in Supplementary Fig. 22.

(b) Western blot shows that not only recombinant ^{myc}(L)^{his} , but also endogenous PLA2g6(L) protein from WT mouse can be specifically recognized by PIN antibody. Blot on the bottom shows the same membrane stained for β-actin. Please, notice that recombinant protein contains myc and his tags on its N and C termini, respectively, which slightly increase its MW in comparison with equivalent endogenous protein in WT mice. Images have been cropped for presentation. Full size images are presented in Supplementary Fig. 22.

(c) Representative TIRF image of MEF cell stained with mPIN ab. TIRF image (the bottom of the cell) shows that PIN ab recognizes endogenous PLA2g6(L) at plasma membrane.

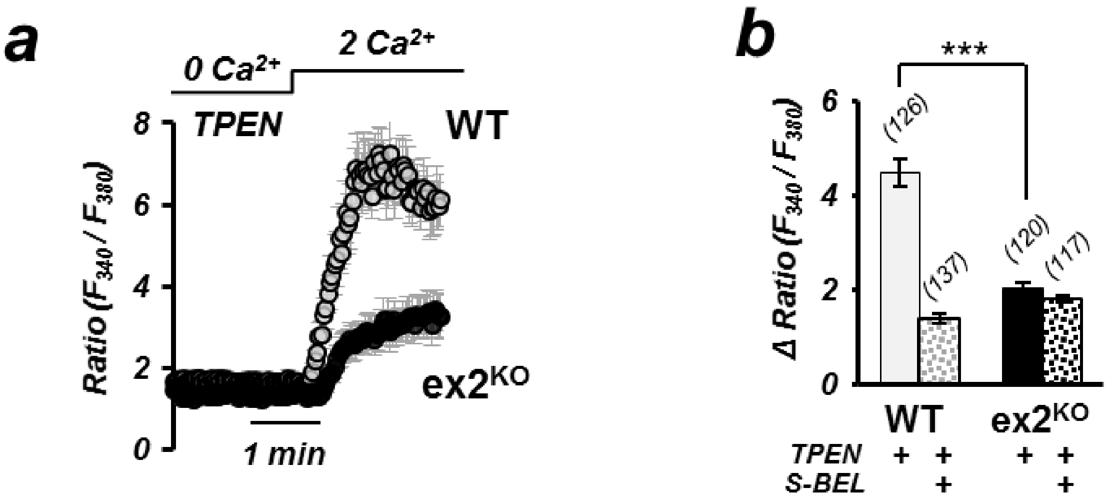

Supplementary Figure 11

Impairment of TPEN-induced PLA2g6-dependent store-operated Ca^{2+} entry (SOCE) in primary mouse embryonic fibroblasts (MEFs) from PLA2g6 ex2^{KO} mice.

(a) Representative traces show Ca^{2+} influx in WT and ex2^{KO} cells pretreated with TPEN (400μM for 5 min). The average Ratio (F_{340}/F_{380}) ±SD is recorded from a group of 10-20 individual cells measured simultaneously. TPEN-induced buffering of Ca^{2+} in ER stores is known to mimic TG-induced depletion of ER, and both treatments activate similar SOCE (as shown in main Figure 1b).

(b) Summary data from experiments show the peak TPEN-induced SOCE in the WT and ex2^{KO} cells in control conditions, and after PLA2g6 inhibition with S-BEL (50μM for 20 min): average ±SE from 3-6 independent experiments per each condition. *** $p<0.001$

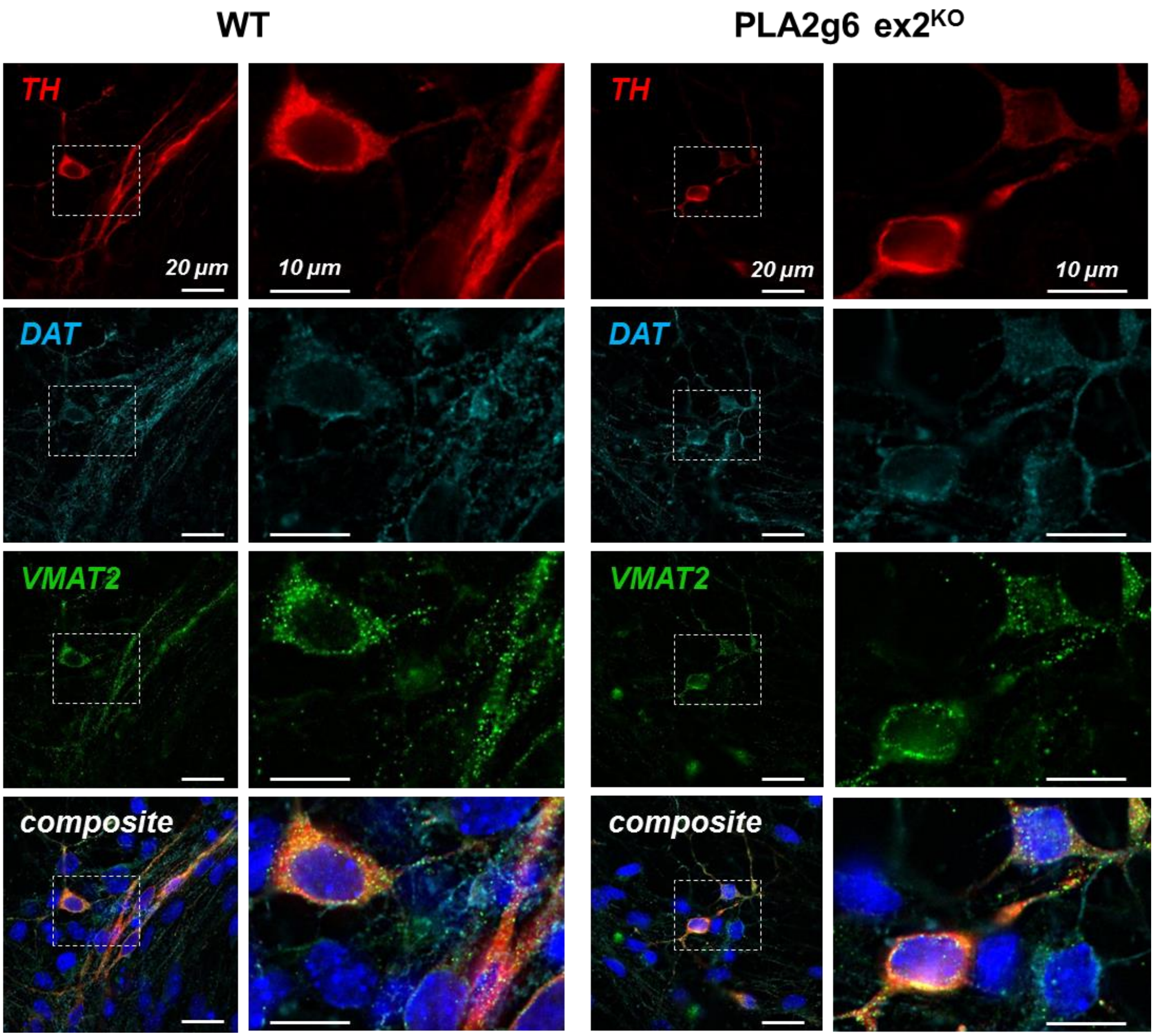

Supplementary Figure 12

Characterization of iPSC-derived TH positive dopaminergic neurons from WT and ex2^{KO} mice.

Representative images show the results of immunostaining of iPSC-derived DA neurons (31 DIV) for tyrosine hydroxylase (TH from Abcam, red), DAT (Abcam, cyan), and VMAT2 (Abcam, green); the nuclei were stained with DAPI (blue). Enlarged images (on the right) focus on individual DA neurons and represent corresponding areas on the less detailed images that show larger area. The composite images show co-localization of TH with other dopaminergic neuronal proteins (DAT and VMAT2) typical for mature DA neurons.

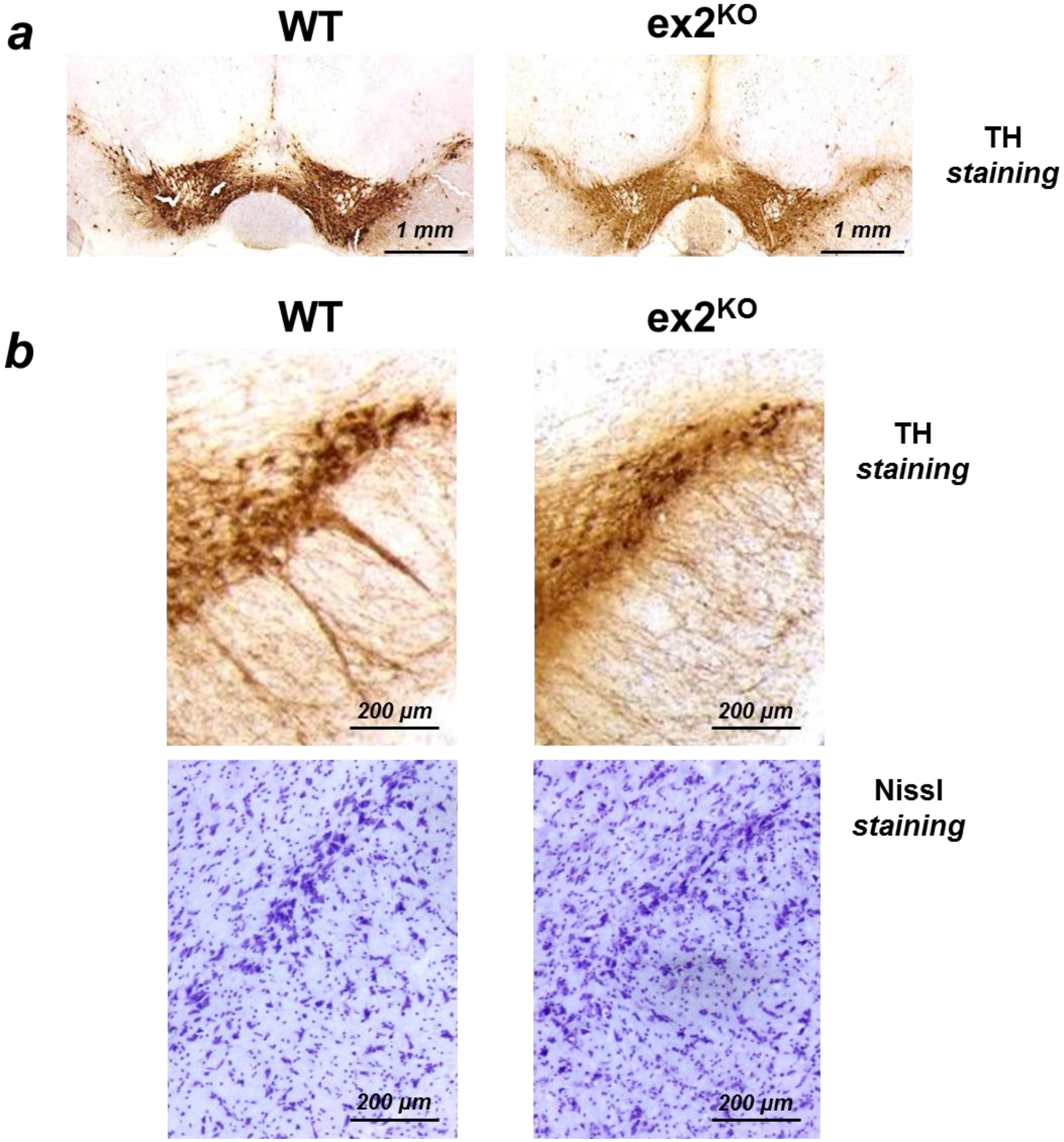

<u>**Supplementary Figure 13**</u>

Representative images of VTA (a) and SNc (b) areas of the brain of WT and ex2^{KO} littermates (24 months old).

(a) Immunostaining for tyrosine hydroxylase (TH, brown) of VTA area.

(b) TH (brown) and Nissl (blue) staining shows significant loss of DA neurons in SNc area of ex2^{KO} mice

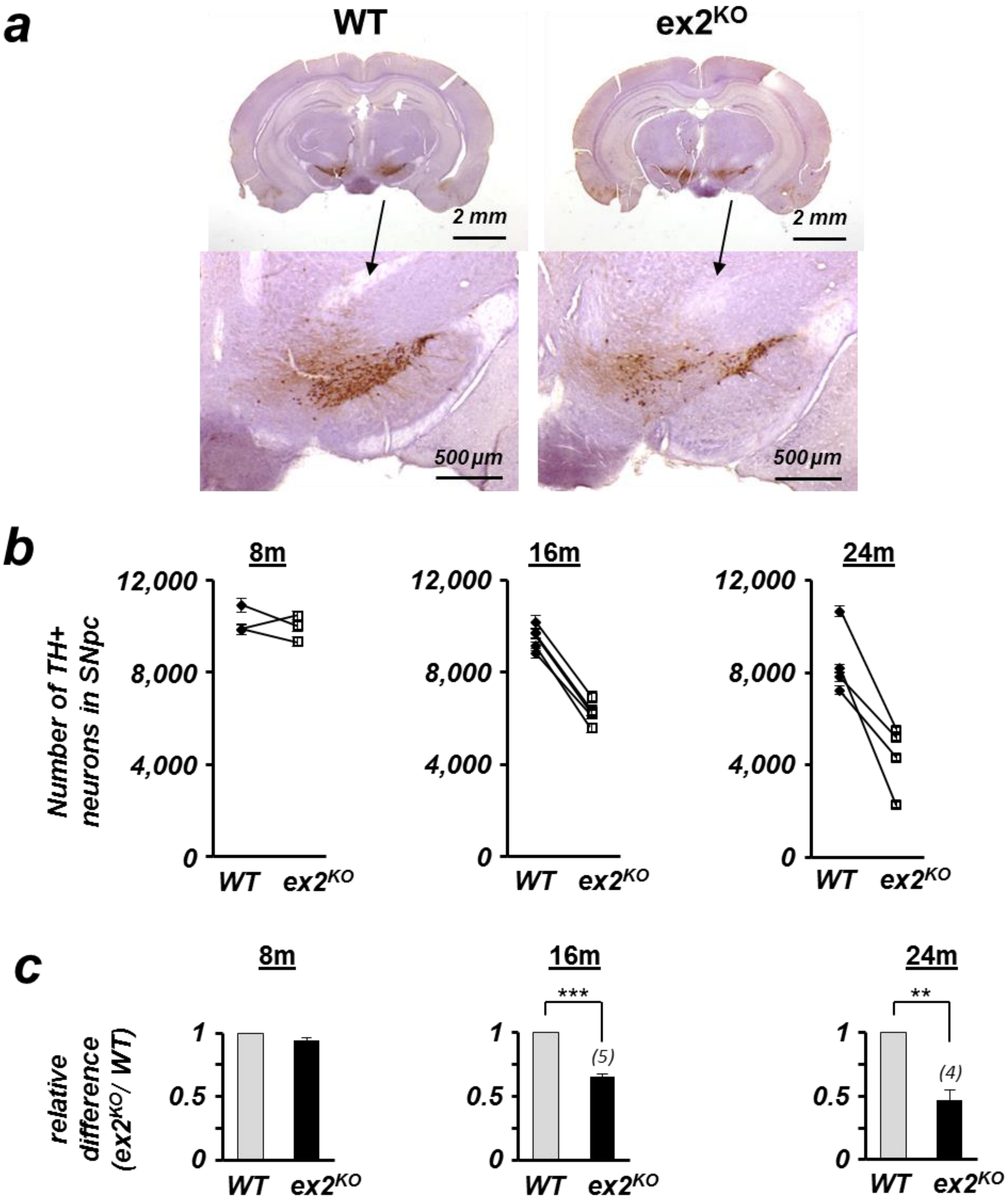

Supplementary Figure 14

Results of blinded stereological analysis of TH+ neurons in SN of 8, 16 and 24-month old WT and ex2^{KO} littermates.

(a) Representative immunostaining for tyrosine hydroxylase (TH from Calbiochem, brown) in brain slices and corresponding (enlarged) nigrostriatal area of the brain of 16-month old WT and ex2^{KO} littermates.

(b) Results of blinded stereological analysis of the total numbers of TH+ neurons in SN (both sides) of WT and ex2^{KO} mice (data for littermate pairs are connected with lines).

(c) Summary data for WT and ex2^{KO} mice show relative differences in the numbers of TH+ neurons in ex2^{KO} animals normalized to their WT littermates. **(p<0.01) ,***(p<0.001).

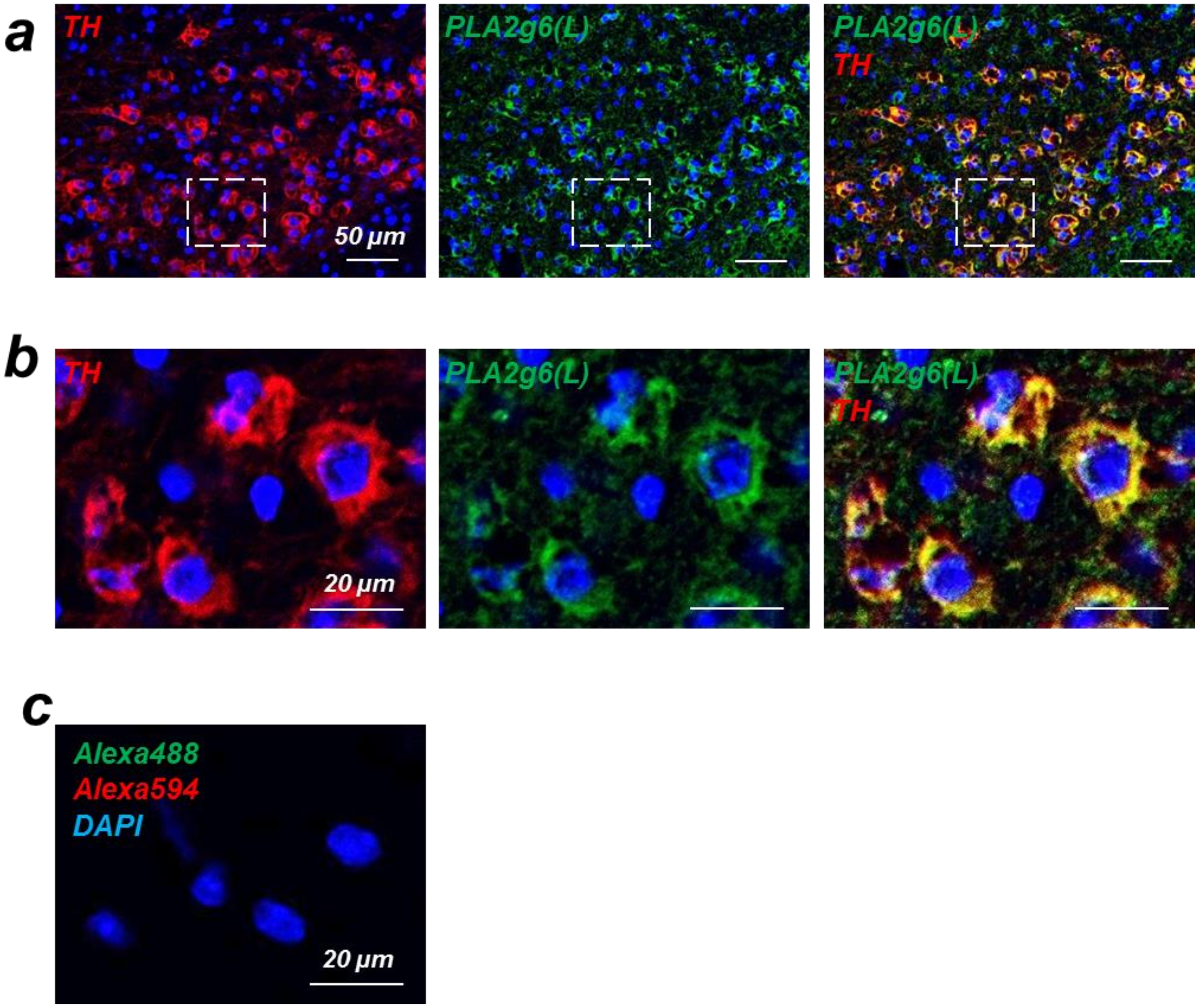

Supplementary Figure 15

High level of PLA2g6(L) protein in dopaminergic TH+ neurons in SNpc of ex2^{KO} mice.

(a) Immunostaining of substantia nigra par compacta (SNpc) area shows localization of tyrosine hydroxylase (TH from Abcam, red) with PLA2g6(L) (custom-made αPIN, green); nuclei stained with DAPI (blue);

(b) Enlarged images of the corresponding areas identified in ***(a)***.

(c) Negative control showing the results of staining with secondary Alexa594 and Alexa488 antibodies (both from Molecular Probes) in the absence of primary antibodies.

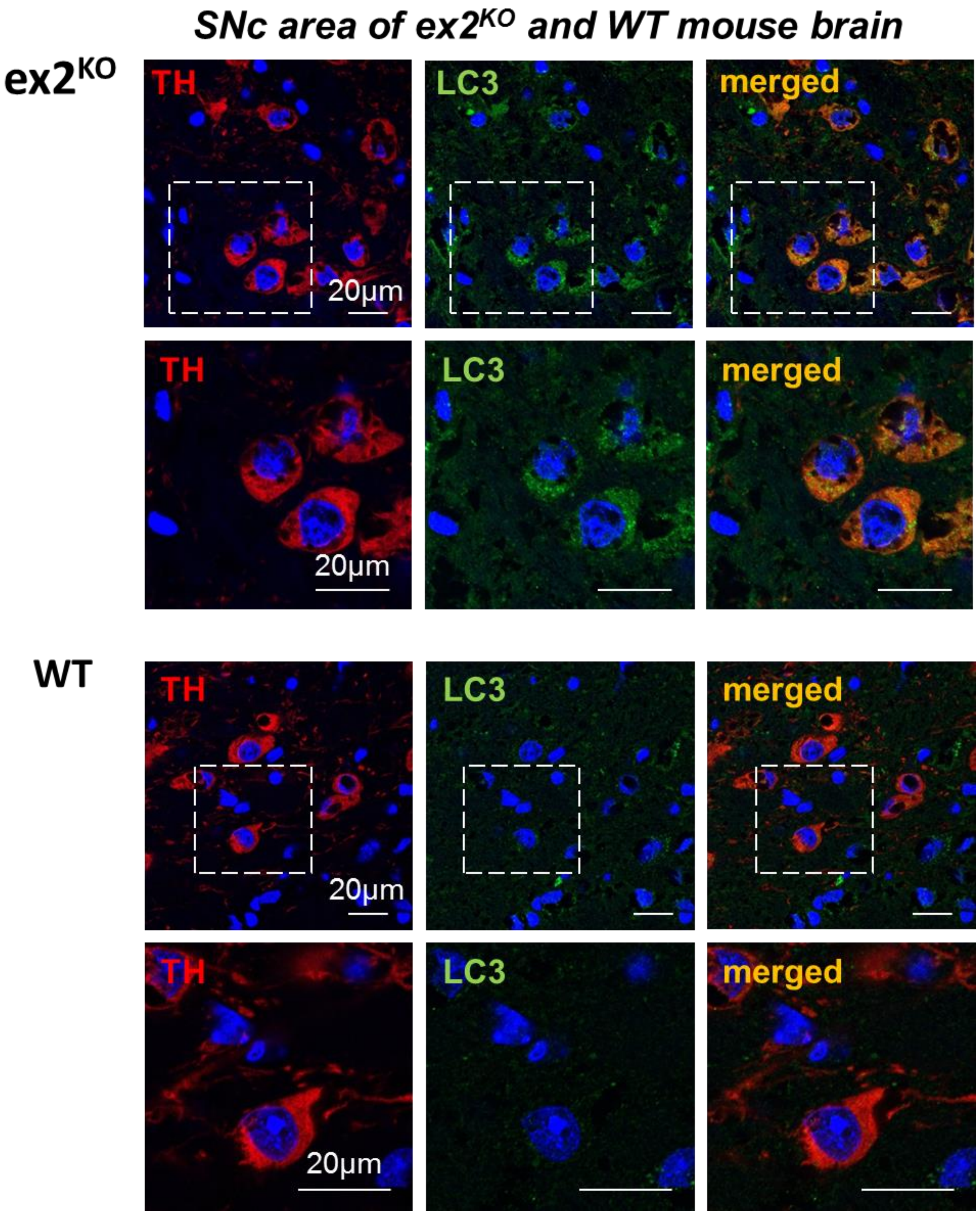

Supplementary Figure 16

Accumulation of endogenous LC3 in TH+ neurons in SNpc of ex2^{KO}, but not WT 16-month old mice.

Representative images show the results of immunostaining for tyrosine hydroxylase (αTH from Abcam, red), LC3 (αLC3B from Cell Signaling, green) and DAPI (blue) of neurons in SNpc area of the brain from WT and ex2^{KO} littermates (16-month old).

Mouse embryonic fibroblasts (MEF) from ex2^{KO} and WT mice

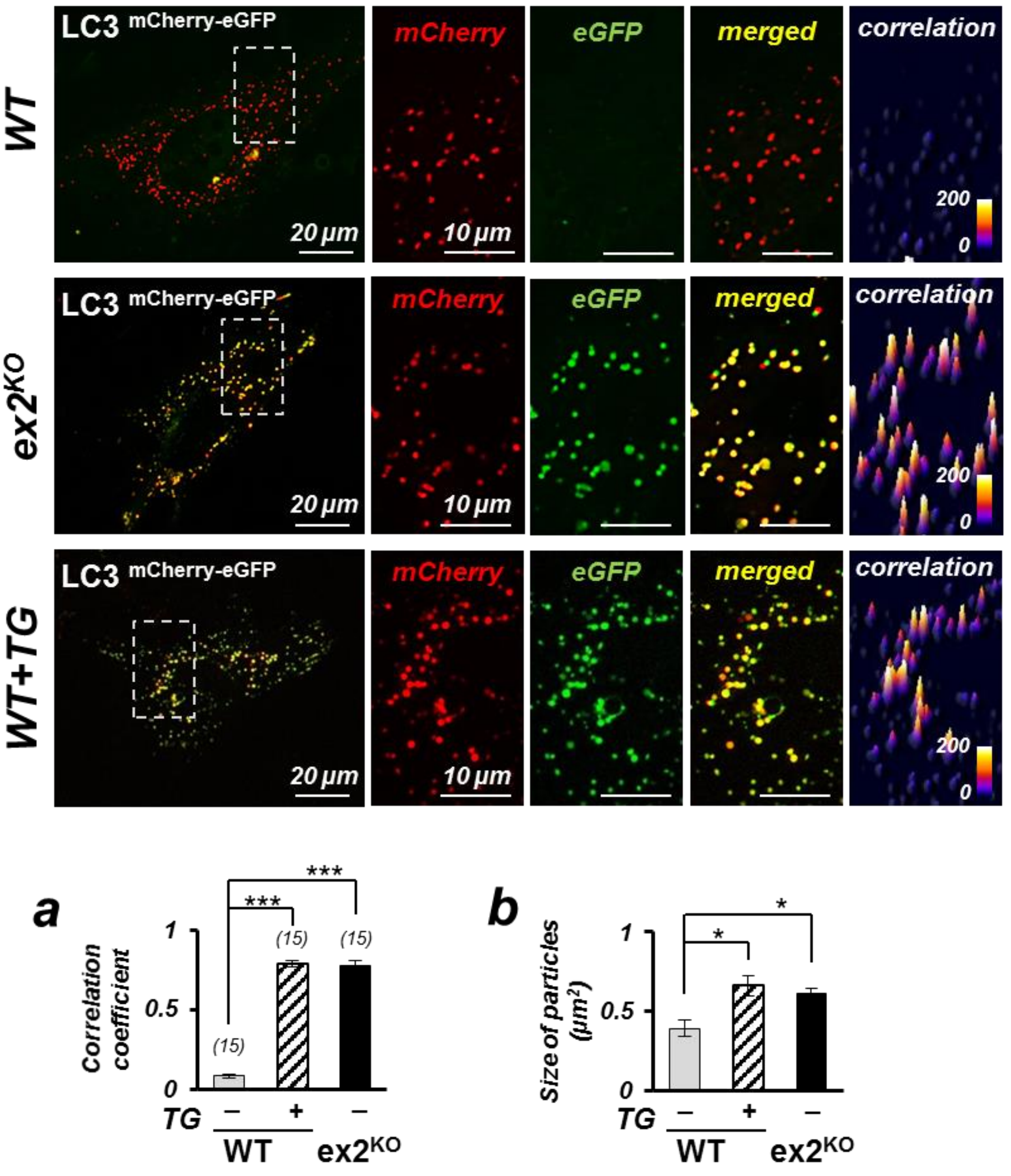

Supplementary Figure 17

Analysis of LC3$^{mCherry/eGFP}$ autophagic flow in WT and ex2^{KO} MEFs, and WT MEFs treated with thapsigargin.

Representative images (the whole cells are shown on the left, and enlarged part of the cells are shown on the right) and correlation maps of tandem mCherry (red) / eGFP(green) tagged LC3 in live MEF cells. Experiments were done 48 hours after transfection. Thapsigargin treatment was 10nM for 24 hours before the experiment. Below are the summary results of comparative analysis of ***(a)*** correlation coefficient of mCherry and eGFP, and ***(b)*** the size of mCherry particles; the data show average ± SE; summary data from a total of 15 cells per condition (5 cells from each of 3 independent experiments), *($p<0.05$), ***($p<0.001$).

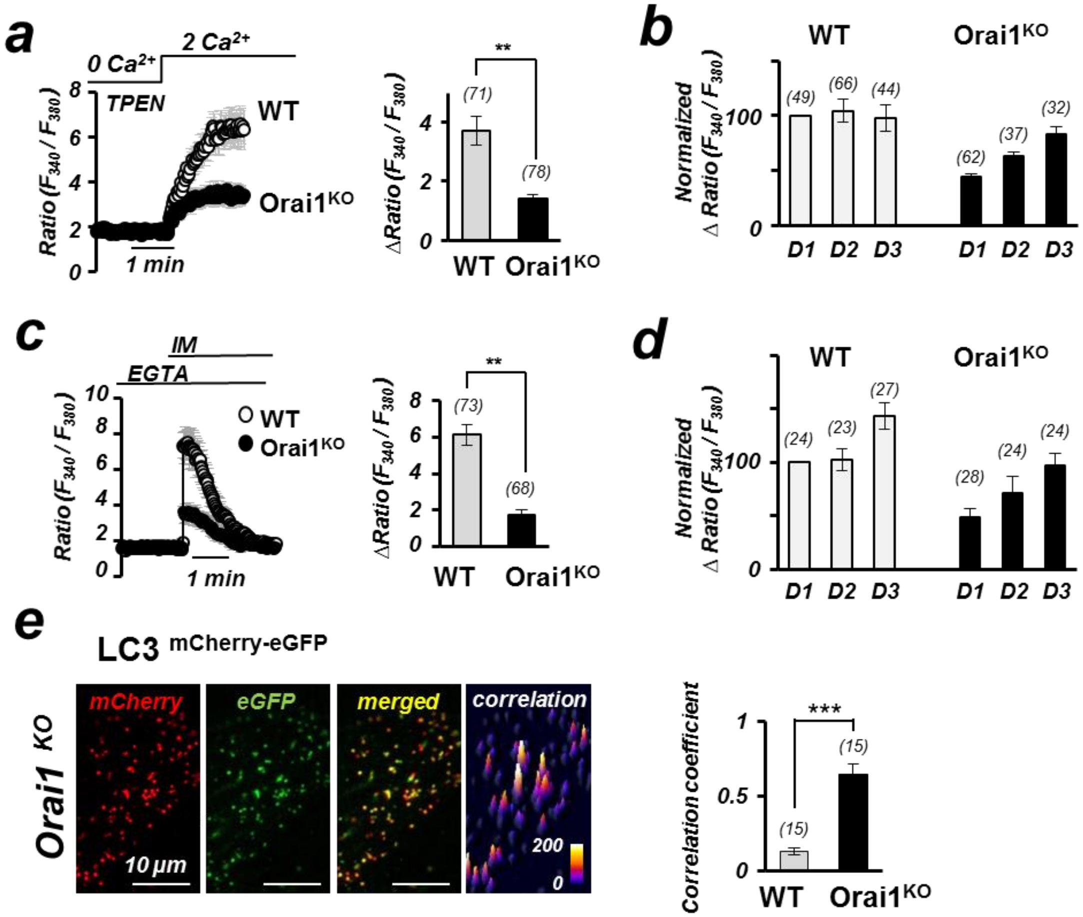

Supplementary Figure 18

Primary MEFs from Orai1 knockout (Orai1^{KO}) mice have impaired SOCE, depleted ER Ca^{2+} stores and significant autophagic dysfunction, which mimic deficiencies found in MEFs from PLA2g6 ex2^{KO} mice.

(a) Representative traces (left) and summary data (right) show significant impairment of TPEN-induced SOCE in MEFs from Orai1^{KO} mice compared to WT control (1 day in culture). Traces show the average SOCE (±SD) from a group of 10-20 individual cells measured simultaneously. Summary data show average (±SE) from 3 independent experiments; the numbers of the cells analyzed in shown above the bars.

(b) Progressive increase in TPEN-induced Ca^{2+} entry following prolonged culture of MEFs from Orai1^{KO} mice demonstrate the ability of MEFs to compensate for genetic Orai1 (and SOCE) deficiency. SOCE was assessed in MEFs (P2) from WT and Orai1^{KO} mice after 1, 2 and 4 days in culture.

(c) Representative traces (left) and summary data (right) show significant loss of ionomycin (IM)-induced Ca^{2+} release from the stores in MEFs from Orai1^{KO} mice (1 day in culture). Traces show the average SOCE (±SD) from a group of 10-20 individual cells measured simultaneously. Summary data show average (±SE) from 3 independent experiments; the numbers of the cells analyzed are shown above the bars.

(d) Progressive increase in IM-induced Ca^{2+} release following prolonged culture of MEFs from Orai1^{KO} mice demonstrate the ability of MEFs to compensate for Orai1 deficiency, and to restore their ER stores.

(e) Representative images (left) and summary data for correlation coefficient (right) of tandem mCherry (red) / eGFP(green) tagged LC3 in live MEF cells from Orai1^{KO} mice. Experiments similar to those described in main Figure 4d,f. The data show average ± SE; summary data from a total of 15 cells per condition (5 cells from each of 3 independent experiments). ** p<0.01***(p<0.001).

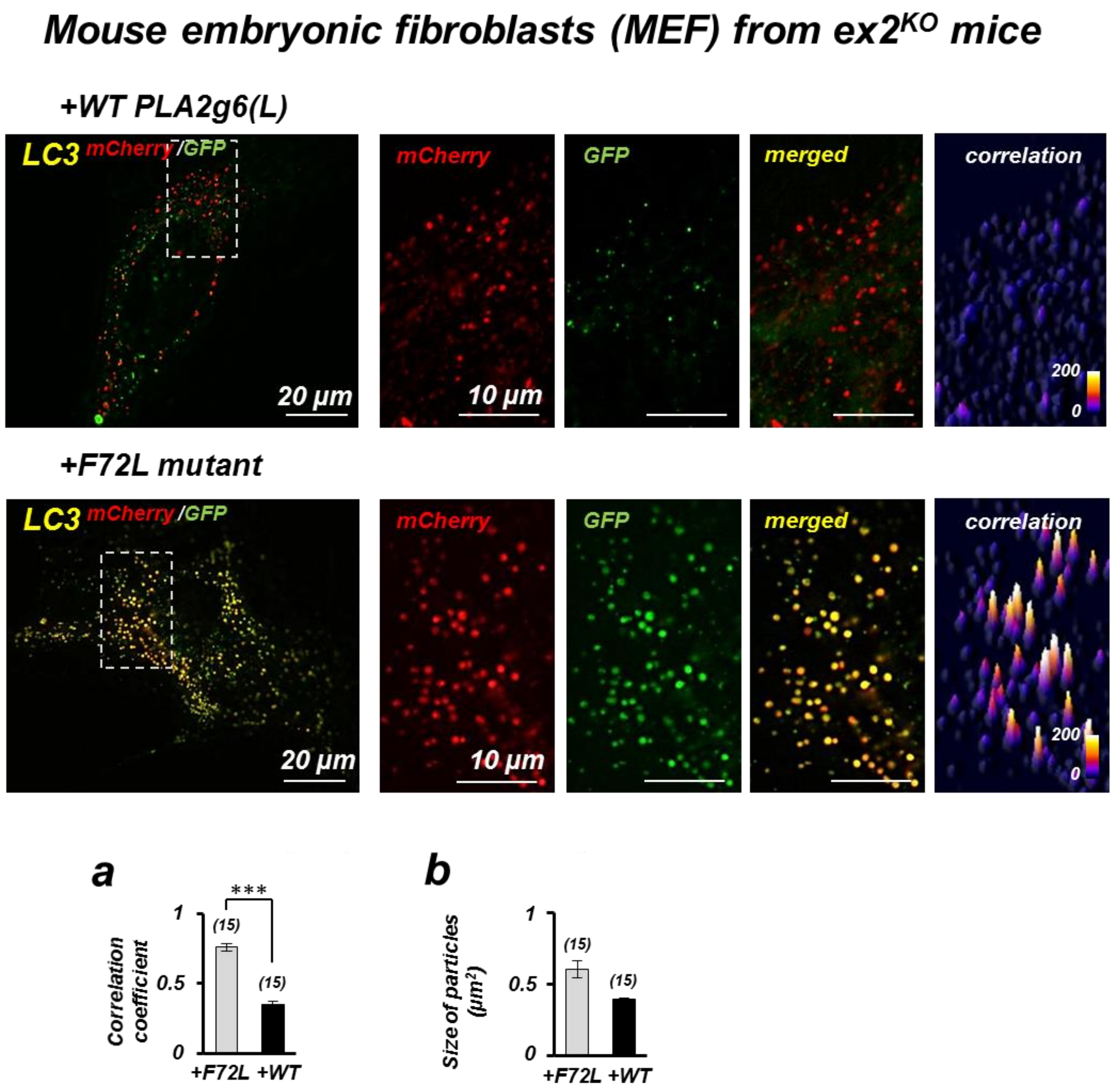

Supplementary Figure 19

Rescue of LC3$^{mCherry/eGFP}$ autophagic flow in ex2^{KO} MEFs by WT PLA2g6(L), but not F72L mutant.

Representative images (the whole cells are shown on the left, and enlarged part of the cells are shown on the right) and correlation maps of tandem mCherry (red) / eGFP(green) tagged LC3 in live MEF cells from ex2^{KO} mice. The cells where transfected with LC3$^{mCherry/eGFP}$ together with either WT PLA2g6(L) (images on the top), or its human PD-associated F72L mutant (images on the bottom). Experiments were done 48 hours after transfection. Below are the summary results of comparative analysis of ***(a)*** correlation coefficient of mCherry and eGFP, and ***(b)*** the size of mCherry particles; the data show average ± SE; summary data from a total of 15 cells per condition (5 cells from each of 3 independent experiments), ***(p<0.001).

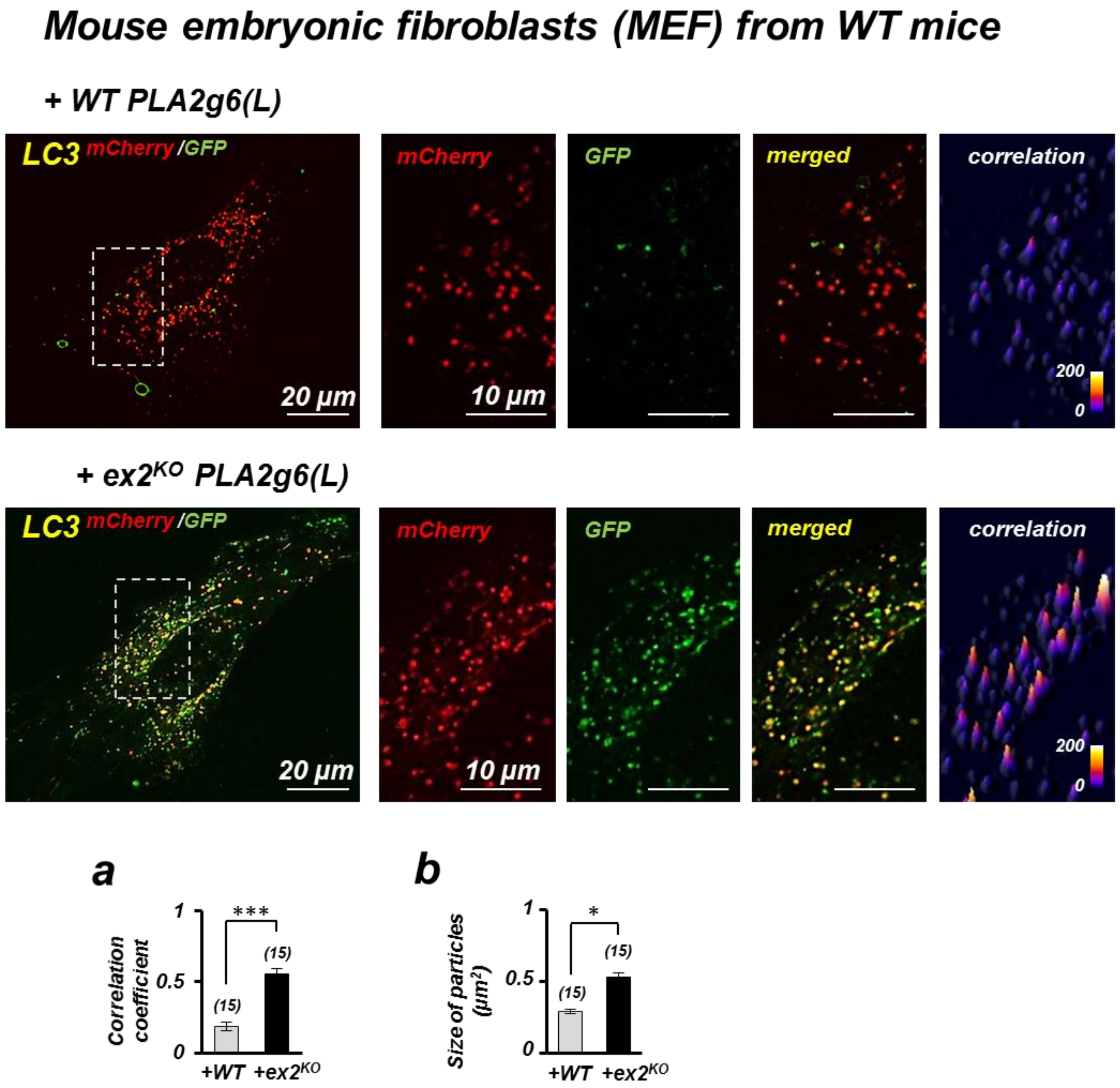

Supplementary Figure 20

Dominant-negative effect of ex2^{KO} PLA2g6(L) on LC3$^{mCherry/eGFP}$ autophagic flow in WT MEFs.

Representative images (the whole cells are shown on the left, and enlarged part of the cells are shown on the right) and correlation maps of tandem mCherry (red) / eGFP(green) tagged LC3 in live MEF cells from WT mice. The cells where transfected with LC3$^{mCherry/eGFP}$ together with either WT PLA2g6(L) (images on the top), or ex2^{KO} (N-terminus truncated) PLA2g6(L) that mimics PLA2g6 protein found in ex2^{KO} mice (images on the bottom). Experiments were done 48 hours after transfection. Below are the summary results of comparative analysis of ***(a)*** correlation coefficient of mCherry and eGFP, and ***(b)*** the size of mCherry particles; the data show average ± SE; summary data from a total of 15 cells per condition (5 cells from each of 3 independent experiments), ***(p<0.001).

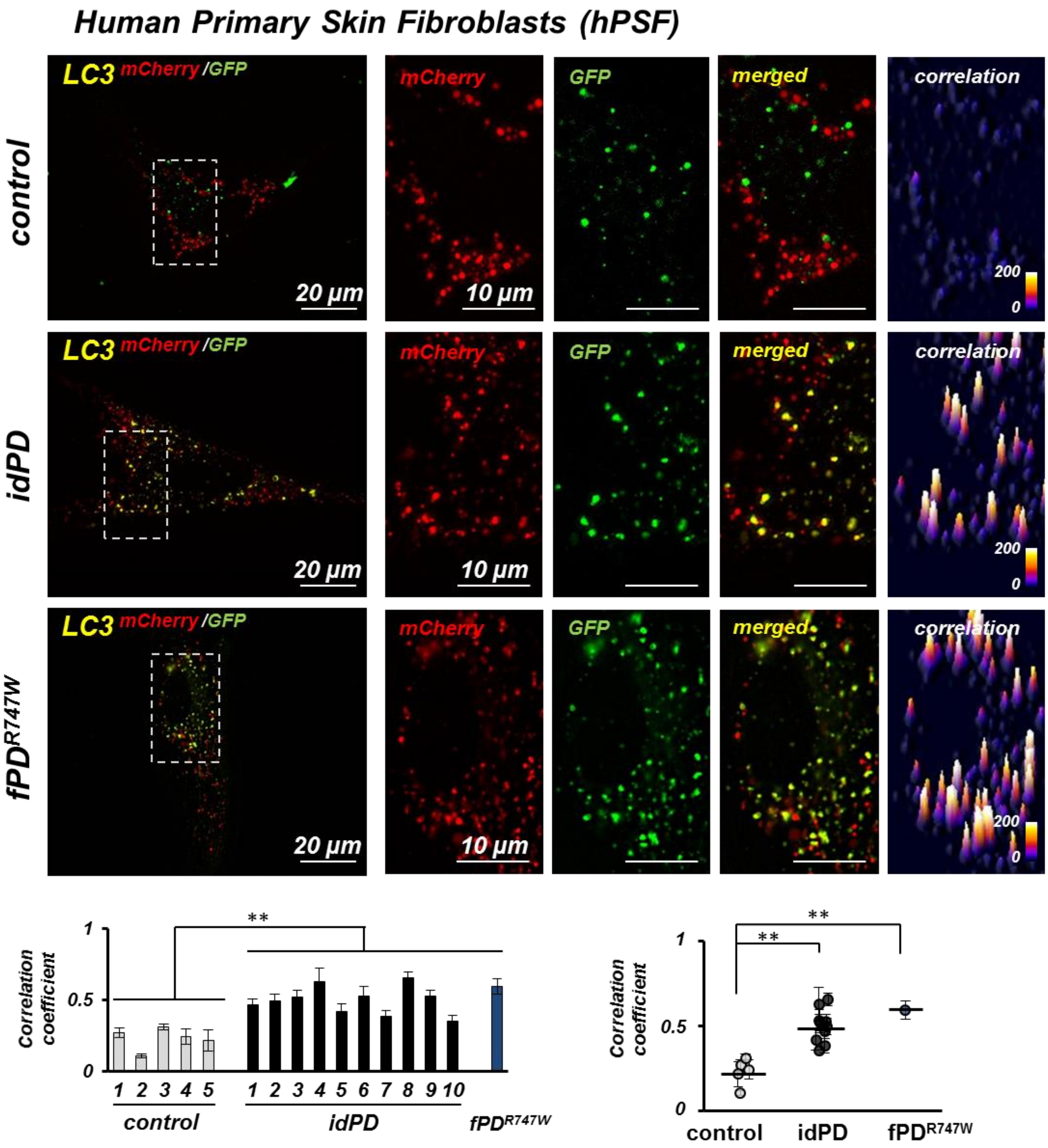

Supplementary Figure 21

LC3$^{mCherry/eGFP}$ autophagic flow in hPSF from control, idPD and fPD^{R747W} patients.

Representative images (the whole cells are shown on the left, and enlarged part of the cells are shown on the right) and correlation maps of tandem mCherry (red) / eGFP(green) tagged LC3 in live hPSF from control, idPD and familial PD (PLA2g6^{R747} mutant) patients. Experiments were done 48 hours after transfection. Below are the summary results of comparative analysis of correlation coefficient of mCherry and eGFP in hPSF from individual patients (left), summary of correlation coefficients for three groups of patients (right): the data show average (± SE) in 15 cells for each patient, **(p<0.01).

Main Figure 5c

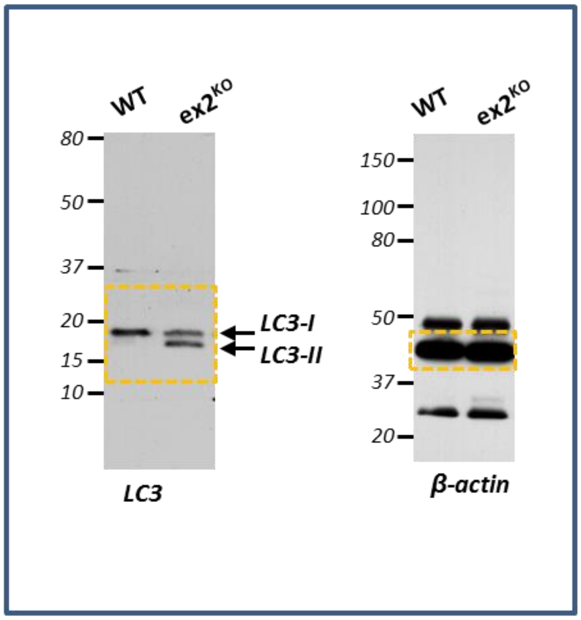

Supplementary Figure 9

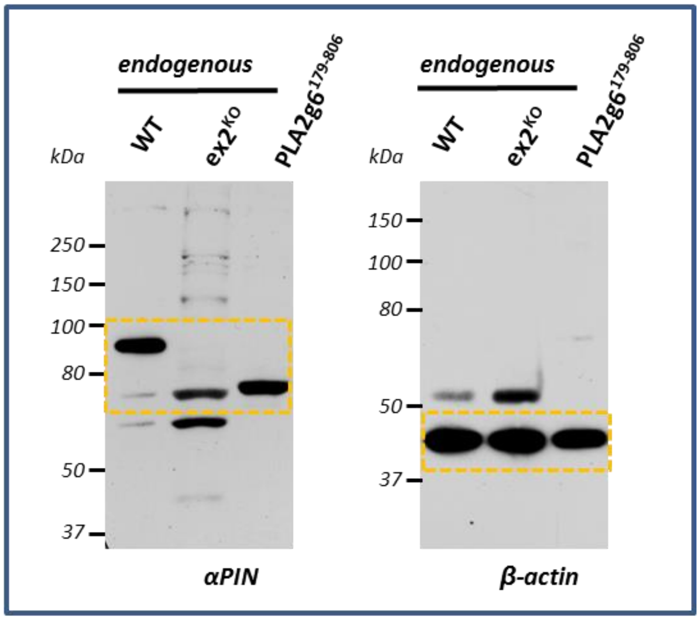

Supplementary Figure 10a

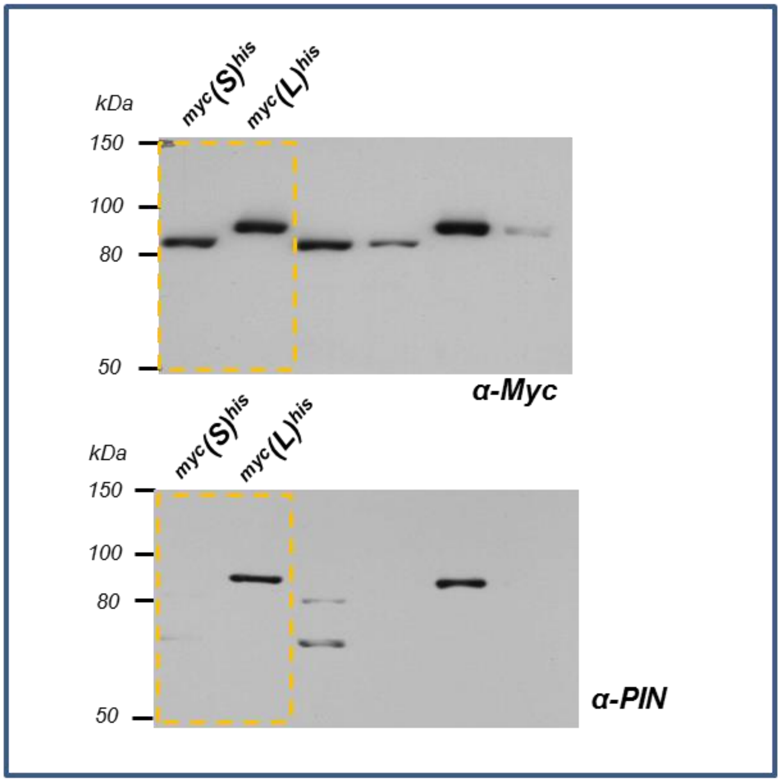

Supplementary Figure 10b

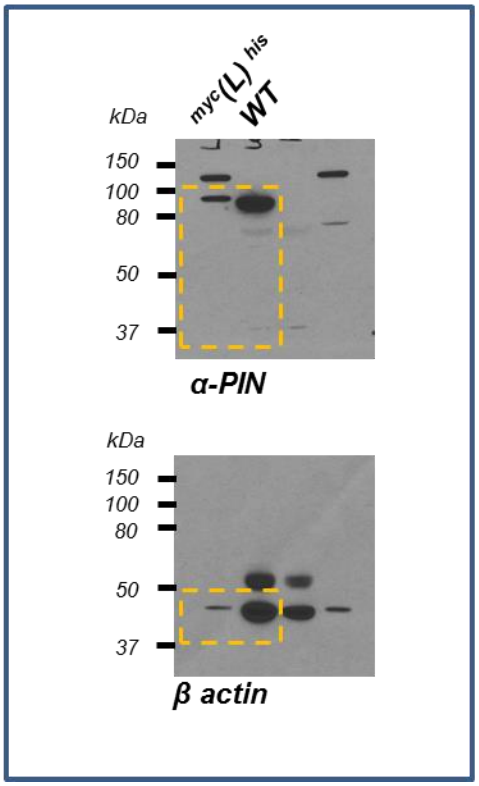

<u>Supplementary Figure 22</u>

Full images of the blots used in Main Figure 5c and Supplementary Figures 9, 10a, and 10b.

Orange rectangles show the parts of the blots that have been cropped for presentation.

a

Sample	Catalog ID	Passage	Onset age	Collection age	Duration
control1	ND34770	P2	--	72	--
control2	ND29179	P3	--	68	--
control3	ND35044	P2	--	77	--
control4	ND29178	P3	--	66	--
control5	ND38530	P5	--	55	--
		average ± SD		68 ± 8	

b

Sample	Catalog ID	Passage	Onset age	Collection age	Duration
idPD1	ND32697	P2	55	58	3
idPD2	ND34265	P2	55	62	7
idPD3	ND39510	P2	50	69	19
idPD4	ND39955	P2	50	55	5
idPD5	ND35976	P2	59	63	4
idPD6	ND33424	P2	47	57	10
idPD7	ND37132	P2	60	66	6
idPD8	ND35320	P1	59	64	5
idPD9	ND37609	P3	62	68	6
idPD10	ND35322	P3	49	61	12
		average ± SD	55 ± 5	62 ± 5	7 ± 5

c

Sample	Catalog ID	Passage	Onset age	Collection age	Duration
PLA2g6-m1	ND32974	P6	18	24	6

Supplementary Table 1

A list of human primary skin fibroblasts (hPSF) used in this study:
(a) 5 control individuals (control),
(b) 10 idiopathic PD (idPD) patients and
(c) patient with familial PD caused by PD-associated R747W mutation in PLA2g6 (fPD $PLA2g6^{R747W}$)
Basic information provided by NINDS Repository (Coriell Institute for Medical Research).

SUPPLEMENTARY METHODS

Animal models.

A novel PARK14 (*Pla2g6*) ex2^{KO} transgenic mouse model (B6.Cg-*Pla2g6*$^{\Delta Ex2\text{-}VB}$/J), in which ex2 of *Pla2g6* gene was constitutively deleted, was custom created by GenOway (France). The strategy is outlined in Supplementary Fig. 5. Briefly, the DNA fragment containing exon 2 and adjacent intron regions of the *Pla2g6 gene* was isolated by PCR from the 129Sv/Pas genetic background mouse, and subcloned into the pCR4-TOPO vector (Invitrogen). To construct a targeting vector, a fragment including exon 2 (containing ATG_1 codon) and a fragment located in the third intron of the *Pla2g6* gene were used to flank a neomycin selection cassette (FRT site-MC1-Neo-FRT site-loxPsite), and a distal loxP site in intron 1. The 129Sv ES cells (GenOway, France) were electroporated with the linearized targeting construct and homologous recombination was assessed in 1408 selected ES cell clones via PCR and Southern blot (Supplementary Fig. 6). One of the *Pla2g6* recombined ES cell clones was microinjected into C57BL/6 blastocysts, and gave rise to male chimeras with significant ES cell contribution as determined by an agouti coat color ≥50%. After mating the chimeras with C57BL/6 female, the agouti colored F1 offspring were genotyped for germ line transmission of the *Pla2g6* recombined allele. Floxed heterozygous *Pla2g6* conditional knockout animals were generated by Flp-mediated excision of the neomycin resistance gene. The heterozygous constitutive ex2 knockout mice were generated by breeding of floxed conditional heterozygous mice with ubiquitous Cre recombinase C57BL/6 mice, and Cre-mediated excision of targeted exon 2 was verified by genotyping of tail DNA via PCR (Supplementary Fig. 7). Oligonucleotides used as PCR genotyping primers were as follows: set 1: gtgaaca-cacaggctaaggctccaatcta and tcaacaagcaaaggacagacatcccac; set 2: agcagaggggcaggctgggtctctc and aggaacacagttgttgggctggggttgtc; set 3: tatcttctcgagttctctagcctccaatcctggg and cacatagaattcgtccccttgca-cagcgtaatgg; and set 4: agcagaggggcaggctgggtctctc and cacatagaattcgtccccttgcacagcgtaatgg.

Heterozygote ex2^{KO} males were backcrossed to C57BL/6 females for 9 generations, and congenic B6.Cg-*Pla2g6*$^{\Delta Ex2\text{-}VB}$/J line was established. Due to infertility of homozygous ex2 knockout males, and the inability of homozygous females to produce/sustain live pups, cross-breeding of heterozygous mice was used to produce homozygous constitutive ex2 knockout (ex2$^{\mathbf{KO}}$) animals that were used in this study. Ageing male mice were used for all live animal studies, while female mice were used for MEF cell preparation. Experimental sets of homozygous ex2 KO (ex2^{KO}) and wild type (WT) littermate males were housed and aged together.

Constitutive Orai1 knockout (Orai1^{KO}) mice (Supplementary Reference [1]) were kindly provided by Dr. Monica Vig. Because homozygous Orai1^{KO} mice have a very limited life span (up to 4-6 weeks), cross-breeding of heterozygous mice was used to produce embryos for Orai1^{KO} MEF cell isolation.

Animal number for each study group was determined for the experimental results to reach statistical significance with a power of 90% at $p<0.05$, or to demonstrate that there is no difference between the groups. Animals were maintained in an advanced pathogen-free facility with veterinary service and unlimited access to food and water. All experimental procedures were compliant with ethical regulations and approved by the Institutional Animal Care and Use Committee of Boston University.

Motor coordination tests.

Age-dependent progression of the overall motor deficit was determined during monthly evaluation of ex2^{KO} and WT mice for the signs and severity of clinical symptoms. Motor deficit was assessed in arbitrary units (AU) using the following scale: 0 = No abnormalities noted; 1 = first subtle signs of motor dysfunction; 2 = clear signs of movement impairment, but sustained postural stability; 3 = obvious signs of impairment in movement and occasional postural instability; 4 = strong ataxia and instability, but no difficulty with eating, drinking and grooming; 5 = very strong ataxia resulting in difficulty with keeping sternal/upright position, and frequently falls when walking, but still able to eat, drink and groom, although with some difficulty. Figure 3a present the time course of motor deficit development in age matched groups of WT and ex2^{KO} mice, and each point indicates median severity ±SE of the symptoms in multiple animals. The number of animals tested at different time points is identified on the graph.

The balance beam test (Supplementary Reference [2, 3]) was used to assess the ability of ageing ex2^{KO} and WT mice to maintain balance while walking along a narrow (2cm) beam placed 20 cm above a soft mattress. Each mouse was placed on a beam for 2 minutes, and its movement was recorded by video camera. The total travel distance, the number of missteps (paw faults, or slips) during travel, and the number of falls off the beam were analyzed for each mouse, and data summarized for each group as mean ±SE. The numbers of animals used for each group are identified on the graphs.

L-DOPA challenge test (Supplementary Reference [3]) was performed on three age groups (12, 16 and 20 months old) ex2^{KO} male mice with motor deficits. Control balance beam test was performed in the morning a day before L-3,4-dihydroxyphenylalanine (L-DOPA) challenge. In the morning of the following day, all mice received a single dose of L-DOPA (5, 10 or 25 mg/kg, Sigma) via peritoneal injection. Twenty minutes before L-DOPA administration, mice were given 6 mg/kg of benserazide (Sigma) to inhibit peripheral DOPA decarboxylase. The balance beam test was done 1 h after L-DOPA injection, as described above. Video-recorded data for each mouse were analyzed later, and summarized for each group as mean ±SE. The numbers of animals used for each age group are identified next to the graph.

The pole test was performed using the standard approach (Supplementary Reference [3]). Briefly, animals were placed head-up on top of a vertical wooden pole (50 cm in length, 1 cm in diameter). The base of the pole was placed in a cage filled with bedding material. When placed on the pole, the animals need to balance on the tip of the pole to orient downward, before they can descend back into the cage. Balance time that is needed to orient downward was analyzed. After 5 training runs, and 1 day resting, each animal received 5 test trials and average of 5 measurements was determined. Summary data show results (mean ±SE). The numbers of animals used for these studies is identified on the graphs.

The rotarod test was performed using the standard approach (Supplementary Reference [2]). Age-matched ex2^{KO} and WT males (16-18 months of age) were tested for the length of time each mouse can maintain its balance and stay on a rotating rod (3 cm diameter). After 4 training sessions and one day resting, the mouse was placed on the rod, and then the rod started to rotate at 5 rpm with acceleration to 40 rpm within 5 minutes until the animal fell from the rod. Average of 4 measurements of the latency to fall (in seconds) was determined for each mouse and data summarized for each group as mean ±SE. The numbers of animals used for these studies is identified on the graphs.

The grip test was performed using the standard approach (Supplementary Reference [2]). Grip Strength Meter (GSM) (Columbus Instruments, Columbus, Ohio) was used to objectively quantify the muscular strength of the forelimbs and hind limbs of age-matched ex2^{KO} and WT animals (16- 18 months of age). All tests (4 repetitions) were performed at the same time in the morning. Strength force was normalized to body weight, which was measured each time after the test. The data were summarized for each group as mean ±SE. The numbers of animals used for these studies is identified on the graphs.

Brain slices: preparation, immunostaining and analysis.

The brains were extracted following paraformaldehyde (PFA, 4% in standard PBS) perfusion, and stored in 4% PFA at 4°C for 24 hours. The brains were then cryopreserved in PBS containing 15% and 30% (w/v) sucrose and stored at 4°C. Brain sections were prepared and stained using standard protocols (Supplementary Reference [4]). Briefly, each brain was embedded and frozen in OCT Tissue-Tek, and a small cut was placed on the right side of the frozen OCT block near the right cortex for side identification. WT and ex2^{KO} brains were sectioned coronal (30μm thickness) with a cryostat microtome and collected as free-floating sections in a 24-well plate and stored at 4°C. The sections containing the substantia nigra pars compacta (SNc) were collected using a staggering method, and six sets of six brain sections were collated: each set contained similar sections from the rostral, middle, and caudal parts of the SNc region. Investigators were blinded during sectioning, TH staining, unbiased stereology and analysis of PAS staining; mouse phenotypes were revealed/confirmed only after all data was generated. No samples were excluded from analysis in targeted age groups.

Blinded unbiased stereological analysis: For stereological analysis (described below) one set of slices from each brain was immunostained with tyrosine hydroxylase (TH) rabbit polyclonal antibody (Calbiochem) and 3,3'-Diaminobenzidine (DAB) HRP substrate (Vector Laboratories) using the standard techniques. Briefly, the endogenous peroxidase activity was blocked by 3% hydrogen peroxide in PBS. The sections were washed with PBS, permeabilized in 0.1% Triton X-100/PBS and blocked with 10% normal goat serum in 0.1% Triton/PBS. The TH antibody was diluted 1:1000 in blocking buffer and incubated overnight at 4°C. The next day, the sections were washed with 0.1% Triton/PBS and incubated in Envision™+ Rabbit (Dako) solution at room temperature for 1 hour. After washing with 0.1% Triton/PBS, DAB (Vector Laboratories) staining was developed. The stained sections were mounted on slides, counterstained with Harris-modified hematoxylin and sealed with Permount. To estimate the number of TH positive (TH+) neurons in SNc of age-matched pairs of ex2^{KO} and WT littermates, matching sets of 6 sections from SNc area of the brain of each experimental animal were analyzed using a Nikon Eclipse E600 microscope and the Stereo-Investigator v11.01.2 software. The total number of TH+ cells in the substantia nigra was estimated using the optical fractionator technique (Supplementary Reference [5]). Briefly, both sides (left/right) of the substantia nigra regions were outlined

separately using a 4X/0.1 air objective (Nikon). Then the TH+ cells were manually counted with a 10X/0.25 air objective (Nikon) using a 60x60 µm counting frame within a 180x180µm grid with a 18µm optical dissector height. 100-200 total cells were counted per section to ensure that the coefficient of error (CE) was less than 0.1. The data were summarized for each group as average ±SE. Paired *t* test was used for statistical analysis of the differences within the matching pairs of WT and ex2^{KO} littermates. The numbers of littermate pairs used for these studies is identified on the graphs.

Periodic acid-Schiff (PAS) staining was performed using the standard procedure and kit available from Sigma (#395B). Briefly, the tissue sections were rehydrated in deionized water and placed in periodic acid solution for 5 minutes. After washing with deionized water, the sections were immersed in Schiff's reagent for 15 minutes. The slides were washed under running tap water for 5 minutes and then counterstained in Gill's Hematoxylin No. 3 for 90 seconds. After a final wash under running tap water, the tissue was dehydrated in alcohol and xylene and mounted. PAS positive cells (stained rose to magenta with blue to black nuclei) were counted in SNc, hippocampus (Hp) and M1/M2 motor cortex regions. Summary data show average number of PAS+ neurons per mm^2 (±SE) from 3 pairs of age-matched WT and ex2^{KO} 16 months old animals.

Immunostaining for TH and LC3 was done using the chicken polyclonal anti-TH antibody (Abcam ab76442), and rabbit polyclonal anti-LC3 antibody (Cell Signaling #2775). Briefly, the free-floating sections were washed with PBS and incubated in 0.1M glycine/PBS for 30 minutes. After additional washing, the sections were transferred to the antigen-retrieval buffer solution (10mM citric acid, 0.05% Tween-20, pH 6.0) and incubated in the steam phase of a 85°C water bath. The sections were then blocked with 10% goat serum/0.1% Triton X-100 for 60 minutes at room temperature. The TH and LC3 antibodies were diluted 1:250 and 1:20 respectively and applied to the sections overnight at 4°C. The following day, the sections were washed and the secondary antibodies goat anti-rabbit Alexa488 (Invitrogen A11034), and goat anti-chicken Alexa594 (Abcam AB150172) were diluted 1:1000 and applied for 60 minutes at room temperature. The nuclei were stained blue with 1ug/ml DAPI (Sigma D9542). Sections were mounted with Vectashield (H-1000) and sealed with nail polish.

Nissl staining (cresyl violet staining) was performed using the standard procedure. Briefly, the tissue was de-fatted for 10 minutes in xylene, followed by 10 minutes in 100% ethanol. The sections were stained with 0.1% cresyl violet acetate (Sigma C5042) solution for 10 minutes and rinsed in tap water to remove the excess stain. After a final 5 minute wash with 80% ethanol, the tissue was cleared in xylene for 5 minutes and mounted with Permount. The Nissl substance in the cytoplasm of neurons stains dark blue and confirms the reduction of TH+ neurons in the substantia nigra.

iPSC-derived A9 midbrain dopaminergic neurons.

Derivation of iPSC from MEFs was performed as previously described (Supplementary Reference [6, 7]). Briefly, ~100,000 MEFs were plated in MEF media and transduced with the constitutive STEMCCA vector at an MOI of 2.5 for 24hr. Media was then changed to ESC media (DMEM supplemented with 15% FBS, 1X GlutaMAX, 350k units of ESGRO leukemia inhibitory factor, and 0.1mM 2-Mercaptoethanol), with media changes every second day until appearance of colonies. Putative iPSC colonies were picked and expanded for STEMCCA excision and characterization. Excision was done as described (Supplementary Reference [8]) using Adeno-Cre infection and confirmed by PCR. Colonies positive for Alkaline Phosphatase, Oct3/4, Zfp96, Nanog and ERas were expanded and banked for neural differentiation. The cultures were routinely checked and confirmed to be negative for mycoplasma.

Differentiation of iPSC into DA neurons was done using standard protocol (Supplementary Reference [9]) by first inducing formation of embryoid bodies in non-adherent conditions for 4 days in knockout serum replacement (KSR) media. Cells were then transferred to adherent plates and incubated in ITS media (DMEM/F12 (Gibco) + 1X ITS Supplement (Sigma I13146) + 1µg/ml Bovine fibronectin (Sigma F1141)) for 6 to 10 days to induce ectoderm formation. Cells were then expanded onto polyornithine/fibronectin coated coverslips in media containing N2 Max supplement, FGF2, FGF-8b, Shh-N and Ascorbic Acid for 5-7 days until cells reach confluency. Neural identity was confirmed by staining against βIII tubulin and nestin. Final differentiation into dopaminergic neurons was done by incubating the cells for 10 more days in minimal media (DMEM/F12 (Gibco) + 1X N2 Max + 200µM Ascorbic Acid (Sigma A4403)). Confirmation of DA neuron identity was done by staining the cells for TH (Abcam ab76442), Dopamine transporter (DAT, Abcam ab5990) and Vesicular monoamine transporter 2 (VMAT2, Abcam 70808).

Confocal and TIRF imaging. Confocal imaging of brain slices was done using LSM710 Duo imaging system (Zeiss, Thornwood, NY,USA) with either a 20x non immersion, or 63X/1.4 Plan-Apochromat oil

immersion objectives, as previously described (Supplementary Reference [11]). Briefly, the 488nm argon laser was used for imaging Alexa 488 and emitted fluorescence was collected in the range of 492-586nm. The 594nm HeNe laser was used to excite Alexa594 and emitted fluorescence was collected in the range of 604-698nm. The 2-photon laser was set to 750nm to visualize DAPI staining and fluorescence was collected in the range of 415-492nm. For better visualization of individual cells, ImageJ was used for background fluorescence subtraction using rolling ball radius of 13.3µm (100 pixels).

Total Internal Reflection Fluorescence (TIRF) imaging of the bottom plane of cells was done using a Nikon Ti inverted fluorescence microscope with a 60x/1.49 Apo-TIRF oil objective (Nikon) and a filter set for GFP illumination.

Western blot. Tissue or cell lysates were prepared with RIPA buffer (50 mM Tris-HCl (pH 7.4), 150 mM NaCl, 1% NP-40, 0.5% sodium deoxycholate, 0.1% SDS, 5 mM EDTA, 5 mM DTT, 1mM PMSF and protease inhibitor cocktail (Roche)). Protein concentrations were determined using a Bio-Rad Bradford protein assay. Proteins (10 µg) were electrophoresed through an 7.5% SDS-polyacrylamide gel for detecting PLA2g6(L) using rabbit polyclonal PIN antibody (1:1000). Proteins (20 µg) were electrophoresed through an 4-20% gradient SDS-polyacrylamide gel for detecting the microtubule-associated protein 1 light chain 3 (LC3) using rabbit polyclonal anti-LC3 antibody (1:1000). Proteins separated by SDS-PAGE were transferred to a Supported Nitrocellulose membrane (Bio-Rad) by electroblotting. The membranes were incubated in blocking buffer (5% nonfat dried milk, 10 mM Tris (pH 7.5), 100 mM NaCl, and 0.1% Tween 20) before immunoblotting was performed with primary Abs. HRP-conjugated anti-mouse or anti-rabbit IgG were used as secondary Ab. The blots were developed with SuperSignal Western Dura Extended Duration Substrate (Thermo Scientific). Western blotting was repeated at least three times.

Quantitative RT-PCR. Total RNA was isolated from hPSF of each individual donor, and from primary MEFs from ex2^{KO} and WT mice using High Pure RNA isolation kit (Roche Applied Science). Concentration and quality of samples was confirmed spectrophotometrically. RNA was reverse-transcribed using High Capacity RNA-to-cDNA Kit (Life Technologies), and cDNA (equivalent of 200 ng RNA) was analyzed per each reaction (in duplicates for technical control) in quantitative PCR on StepOnePlus™ Real Time PCR System (Applied Biosystems).

The following TaqMan® gene expression assays were used for hPSF: Hs00385627_m1 for Orai1, Hs00963373_m1 for STIM1, Hs00957788_m1 for STIM2, Hs00608195_m1 for TRPC1, Hs00899715_m1 for PLA2g6(L), Hs00895670_m1 for PLA2g6(S), and 4333764F for glyceraldehyde-3-phosphate dehydrogenase (GAPDH).

The following TaqMan® gene expression assays were used for mouse cells and tissue: Mm03929082_m1 for PLA2g6(L), Mm03010833_m1 for PLA2g6(S), and 4352932 for mouse glyceraldehyde-3-phosphate dehydrogenase (GAPDH). The relative expression level for each gene to the level of GAPDH in the same sample was analyzed using the comparative C_T method[12]

DNA constructs. Tandem LC3$^{mCherry/cGFP}$ construct was from Addgene. cDNA for human PLA2g6(L) variant (Supplementary Reference [13, 14]) (Genbank #AF064594) was a kind gift from Dr. Brian P. Kennedy (Karolinska Institute, Stockholm, Sweden) and MERCK FROSST CANADA Inc., and was used for creation of all other constructs. His-tagged and/or myc-tagged expression constructs of PLA2g6(L) were created by PCR-subcloning of the full-length long variant of human PLA2g6(L) into pcDNA3.1 plasmid with polylinker between restriction sites *Nhe*I and *Pme*I exchanged for the following sequence: gct agc gtt aac acc ggt atg gaa ttc *gaa caa aaa ctc atc tca gaa gag gat ctg* gat atc cct gca ggc taa gga tcc cac gtg ctc gag cgt ctc caa ttg gcg gcc gca aga gga tcg *cat cac cat cac cat cac* tag agt gaa gct taa gtt taa ac, allowing for expression of various double- and single-tagged fusions with myc and/or his tags . F72L and A80T mutants of ^{myc}PLA2g6(L)^{his} were created by Mutagenex (USA), and confirmed by sequencing.

Supplementary References:

1. Vig,M. *et al.* Defective mast cell effector functions in mice lacking the CRACM1 pore subunit of store-operated calcium release-activated calcium channels. *Nat. Immunol.* **9**, 89-96 (2008).

2. Brooks,S.P. & Dunnett,S.B. Tests to assess motor phenotype in mice: a user's guide. *Nat Rev Neurosci* **10**, 519-529 (2009).

3. Fleming,S.M. *et al.* Behavioral effects of dopaminergic agonists in transgenic mice overexpressing human wildtype alpha-synuclein. *Neuroscience* **142**, 1245-1253 (2006).

4. Jackson-Lewis,V. & Przedborski,S. Protocol for the MPTP mouse model of Parkinson's disease. *Nat. Protocols* **2**, 141-151 (2007).

5. Giannaris,E.L. & Rosene,D.L. A stereological study of the numbers of neurons and glia in the primary visual cortex across the lifespan of male and female rhesus monkeys. *J. Comp Neurol.* **520**, 3492-3508 (2012).

6. Sommer,C.A. & Mostoslavsky,G. Experimental approaches for the generation of induced pluripotent stem cells. *Stem Cell Res Ther* **1**, 26 (2010).

7. Sommer,C.A. *et al.* Induced pluripotent stem cell generation using a single lentiviral stem cell cassette. *Stem Cells* **27**, 543-549 (2009).

8. Sommer,C.A. *et al.* Excision of reprogramming transgenes improves the differentiation potential of iPS cells generated with a single excisable vector. *Stem Cells* **28**, 64-74 (2010).

9. Lee,S.H., Lumelsky,N., Studer,L., Auerbach,J.M., & McKay,R.D. Efficient generation of midbrain and hindbrain neurons from mouse embryonic stem cells. *Nat Biotechnol.* **18**, 675-679 (2000).

10. Kirber,M.T., Chen,K., & Keaney,J.F., Jr. YFP photoconversion revisited: confirmation of the CFP-like species. *Nat. Methods* **4**, 767-768 (2007).

11. Schafer,C., Rymarczyk,G., Ding,L., Kirber,M.T., & Bolotina,V.M. Role of molecular determinants of store-operated Ca(2+) entry (Orai1, phospholipase A2 group 6, and STIM1) in focal adhesion formation and cell migration. *J. Biol. Chem.* **287**, 40745-40757 (2012).

12. Schmittgen,T.D. & Livak,K.J. Analyzing real-time PCR data by the comparative C(T) method. *Nat Protoc.* **3**, 1101-1108 (2008).

13. Larsson Forsell,P.K.A., Kennedy,B.P., & Claesson,H.E. The human calcium-independent phospholipase A2 gene: Multiple enzymes with distinct properties from a single gene. *Eur J Biochem* **262**, 575-585 (1999).

14. Larsson,P.K., Claesson,H.E., & Kennedy,B.P. Multiple splice variants of the human calcium-independent phospholipase A2 and their effect on enzyme activity. *J Biol. Chem.* **273**, 207-214 (1998).
